# Sensory score prediction and key aroma compounds characterization in fermented chopped pepper

**DOI:** 10.1016/j.fochx.2025.102743

**Published:** 2025-07-05

**Authors:** Yuan Liu, Lingyan Zhao, Chunya Yang, Yeyou Qin, Li Zhu, Fangming Deng

**Affiliations:** a College of Food Science and Technology, Hunan Agricultural University, Changsha, Hunan 410128, PR China; b Hunan Tantanxiang Food Technology Co., Ltd, Changsha, Hunan 410128, PR China

**Keywords:** Fermented chopped pepper, Prediction, Machine learning methods, Odor activity values, Key aroma compounds, Linalool (PubChem CID: 6549), Phenethyl alcohol (PubChem, CID: 6054), Methional (PubChem, CID: 18635), 3-Isobutyl-2-methoxypyrazine (PubChem, CID: 32594), Ethyl trans-4-decenoate (PubChem, CID: 5362583), β-Ionone (PubChem, CID: 638014), Spiroxide (PubChem, CID: 61953), Ethyl 2-methylbutyrate (PubChem, CID: 24020), α-Terpineol (PubChem, CID:17100), 4-Ethylphenol (PubChem, CID: 31242)

## Abstract

Fermented chopped pepper (FCP) exhibits complex and variable aroma profiles, making it challenging to accurately predict its sensory scores and identify key aroma compounds. In this study, electronic nose (*E*-nose) combined with machine learning methods were applied for the prediction of FCPs sensory scores. The random forest (RF) demonstrated the highest predictive accuracy among support vector machine (SVM), multiple linear regression (MLR), and back propagation neural network (BPNN). *E*-nose combined with the trained RF was used to predict the sensory scores of FCPs from eight regions. Totally, 97 volatile compounds and 19 odor-active compounds were detected by GC × GC-O-Q-TOF-MS in the top-performing sample (FCP-1). Among these, 34 compounds exhibited odor activity values (OAV) greater than 1. Aroma recombination and omission experiments confirmed that linalool, phenethyl alcohol, methional, 3-isobutyl-2-methoxypyrazine, ethyl trans-4-decenoate, β-ionone, spiroxide, ethyl 2-methylbutyrate, α-terpineol, 4-ethylphenol, β-damascenone, and nerolidol were the key aroma compounds in FCP-1.

## Highlights

• Four machine learning models predicted sensory scores of fermented chopped peppers.
• GC × GC-Q-TOF-MS identified 97 volatiles in FCP-1, with 34 having OAV > 1.
• Esters, aldehydes, pyrazine, etc., were the main volatile compounds in FCP-1.
• 12 key aroma compounds were identified through recombination and omission tests.

## Graphical abstract

**Figure:**
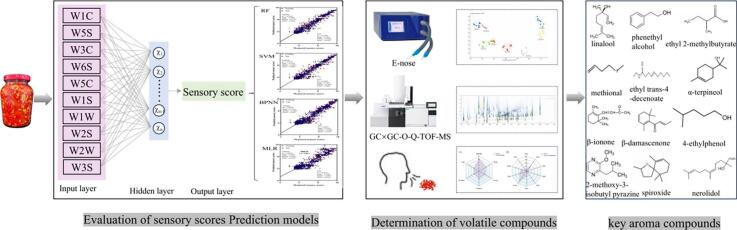

## 1 Introduction

Fermented chopped pepper (*Capsicum annuum L)* is a traditional Chinese seasoning appreciated for its distinctive aroma. Volatile compounds are generally associated with the aroma of foods and are critical determinants of consumer acceptance and market value (Liu et al., 2024). The formation of fermented chopped pepper (FCP) aroma compounds is influenced by raw materials, production methods, and microbial communities, resulting in diverse volatile compounds such as esters, alcohols, terpenes, and other volatiles (Xu et al., 2021). Traditionally, the evaluation of FCP aroma complexity typically relies on human sensory methods, which can directly reflect product quality but are often time-consuming and susceptible to individual biases (Prescott, 2017). Given the intricate nature of its aroma profile and the limitations of traditional sensory methods, there is a growing need for efficient and objective approaches to evaluate sensory quality and comprehensively characterize its volatile composition.

Compared with traditional sensory evaluation, objective analytical methods provide higher standardization and precision. In addition to gas chromatography combined with corresponding pre-treatment technology, electronic nose (e-nose) has been widely applied as a rapid, convenient, and non-destructive tool for characterizing overall aroma profiles while minimizing human influence (Omatu & Yano, 2016). With the invention, development, and application of *E*-nose, significant progress has been made in the study of pepper flavor. For instance, Yan et al. (2025) and Zhang et al. (2024) utilized e-nose to analyze the flavor characteristics of pepper, revealing that esters, alcohols, aldehydes, and other compounds were present in high abundance. However, the key challenge is how to effectively integrate subjective sensory data with objective chemical data. Wijaya et al. (2024) successfully predicted the organoleptic score of Gambung green tea by combining e-nose data with the k-nearest neighbor algorithm. Similarly, Jiang et al. (2023) applied intelligent sensory technologies integrated with the back propagation neural network to predict the sensory attributes of braised beef.

Volatile compounds in FCP primarily include esters, terpenes, alcohols, aldehydes, and pyrazines (Xu et al., 2021). Gas chromatography-mass spectrometry (GC–MS) is the most commonly used instrumental for analyzing compounds. However, owing to the complexity of the FCP aroma profile, more advanced approaches with high-resolution and high-sensitive are required to achieve a more comprehensive and accurate characterization of volatile compounds. Fortunately, two-dimensional gas chromatography coupled with quadrupole time-of-flight mass spectrometry (GC × GC-Q-TOF-MS) has emerged as a powerful tool for in-depth flavoromics analysis, providing superior separation efficiency and high sensitivity to detect trace-level volatiles (Zou et al., 2023). For instance, Guan et al. (2024) found that GC × GC-Q-TOF-MS identified 1026 volatile compounds in cherry tomato, 363 more than detected by GC–MS, which highlighted the analytical advantages of GC × GC-Q-TOF-MS. Furthermore, a comprehensive understanding of the key aroma compounds in FCP were systematically characterized by integrating odor activity value (OAV) calculation with aroma recombination and omission experiments to validate their sensory contributions. Yang et al. (2022) reported that volatile compounds with OAV ≥ 1 were known as key aroma compounds, while volatile compounds with OAV < 1 may exhibit masking or inhibitory to alter aroma perception. Li, Cheng, et al. (2022) identified 70 volatile compounds from fermented bamboo shoots, of which only 7 were shown to be the key aroma compounds using OAV, aroma recombination and omission experiments.

To address this knowledge gap, this study aims to achieve the following objectives: (1) construct sensory score prediction models for FCP using random forest (RF), support vector machine (SVM), multiple linear regression (MLR), and back propagation neural network (BPNN); (2) evaluate and compare model performance based on the correlation coefficient (R^2^), mean squared error (MSE), root mean squared error (RMSE), and mean absolute error (MAE); (3) comprehensively characterize the volatile compounds of FCP-1 by GC × GC-O-Q-TOF-MS; and (4) identify the key aroma compounds through OAV calculation combined with aroma recombination and omission experiments. This study is expected to provide valuable insights for understanding the flavor composition of FCP and offer a reference for its quality control and industrial-scale production.

## 2 Materials and methods

### 2.1 Materials

Eight varieties of fresh peppers from different regions (FCP-1, FCP-2, FCP-3, FCP-4, FCP-5, FCP-6, FCP-7, and FCP-8, as shown in **Table S1**) were individually washed, drained, chopped, and mixed with 10 % (*w*/w) edible salt (NaCl). Subsequently, the mixtures were fermented under consistent conditions in sterilized jars at room temperature for 60 days. The samples were stored at −80 °C until flavor analysis.

### 2.2 Chemicals

To identify and quantify aroma compounds, the following standards were purchased from Macklin (Shanghai, China): 3-methyl-1-butanol (99 %), 4-methyl-1-pentanol (98 %), ethyl 2-methylbutyrate (98 %), hexanol (98 %), isoamyl acetate (99 %), styrene (99 %), methional (98 %), ethyl isohexanoate (99 %), octanal (99 %), hexyl acetate (99 %), β-limonene (95 %), ethyl 2-hexenoate (99 %), phenylacetaldehyde (95 %), guaiacol (99 %), linalool (98 %), nonanal (98 %), phenylethyl alcohol (98 %), 4-ethylphenol (98 %), 3-isobutyl-2-methoxypyrazine (98 %), α-terpineol (96 %), methyl salicylate (99 %), ethyl caprylate (99 %), phenethyl acetate (99 %), ethyl 2-hydroxybenzoate (99 %), spiroxide (90 %), 4-vinylguaiacol (98 %), ethyl (4E)-4-decenoate (98 %), ethyl isovalerate (99 %), β-damascenone (99 %), β-ionone (90 %), nerolidol (97 %), ethyl laurate (99 %) and methanol (99.9 %). 1,2-dichlorobenzene (99 %) and C_7_–C_40_ n-alkane (≥99.9 %, GC) were bought from Sigma-Aldrich (St. Louis, MO). All chemicals used were of analytical grade, unless otherwise stated.

### 2.3 Mathematical models

In this study, RF, SVM, MLR and BPNN were used to develop predictive models for establishing the relationship between e-nose sensor responses and sensory scores of FCPs. The dataset was randomly divided into a training set (70 %) and a test set (30 %) (as shown in **Fig. S1**, **Table S2**). The training set was used to construct and optimize the models, while the test set was used to evaluate the generalization performance on unseen data. The e-nose sensor (W1C, W5S, W3C, W6S, W5C, W1S, W1W, W2S, W2W, and W3S) responses were used as the model inputs, and the sensory score was used as the model output. Prior to model training, the input data were normalized to a [0,1] range using min-max normalization to eliminate inter-features scale disparities and improve model stability. Grid search was applied to optimize model parameters, thereby reducing the risk of overfitting. Eq. (1) was used to perform normalization (Huang et al., 2022):

(1) $$Q_{n}=\frac{Q-Q_{\min}}{Q_{\max}-Q_{\min}}$$

Where$\;Q_{n}$ is the normalized response value of each e-nose sensor, Q is the original value, $Q_{\min}\;$and $Q_{\max}\;$are the minimum and maximum response value in the dataset, respectively.

#### 2.3.1 RF model

RF is an ensemble learning to handle classification and regression tasks effectively. The final prediction in the RF model was calculated by averaging the outputs of all decision trees, as shown in Eq. (2):

(2) $$\hat{y}=\frac{1}{B}\sum_{b=1}^{B}f_{b}\left(X\right)$$

Where $\hat{y}$ is the final predicted sensory score, B is the total number of trees in the forest, $f_{b}\left(X\right)$ is the prediction made by the b-th decision tree for the input X, and X represents the response value obtained from the e-nose sensor.

#### 2.3.2 SVM model

SVM is a supervised learning algorithm used for classification and regression tasks in high-dimensional spaces. The SVM model employed a radial basis function (RBF) kernel to implicitly the e-nose data into a high-dimensional feature space, enabling nonlinear classification. The kernel function was defined as shown in Eq. (3):

(3) $$k\left(x,y\right)=\exp\left(\frac{-{\left\|x-y\right\|}^{2}}{\sigma^{2}}\right)$$

Where x and y are the input vectors representing e-nose sensor response values, $k\left(x,y\right)$ is the kernel function indicating the similarity, exp is the exponential function, and σ is the kernel function parameter controlling the width of Gaussian function.

#### 2.3.3 BPNN model

BPNN is a commonly used multi-layer feed-forward neural network. The hidden layer's activation function was essential for BPNN as it provided the necessary nonlinear modeling capability. In this study, the hyperbolic tangent sigmoid (tansig) function was employed as the activation function, as shown in Eq. (4):

(4) $$\mathrm{tansig}\left(x\right)=\frac{2}{1+e^{-2x}}-1$$

Where, x is the input to the activation function, and $\mathrm{tansig}\left(x\right)$ is the output after non-linear transformation.

#### 2.3.4 MLR model

MLR could be used to predict the relationship between a target variable and two or more feature variables. To model the relationship between the predicted sensory scores and the e-nose sensor responses, we employed Eq. (5) for MLR model:

(5) $$y=\beta_{0}+\beta_{1}x_{1}+\ldots +\beta_{n}x_{n}+c$$

Where y is the predicted sensory score,$\;\beta_{0}$ is a intercept term, $\beta_{n}$ is the regression coefficient corresponding to the n-th sensor, $x_{n}$ is the response value of n-th e-nose sensor, and c is the error term representing unexplained variance or model noise.

#### 2.3.5 Performance criteria

The performance of the RF, SVM, MLR, and BPNN models was evaluated using R^2^ (Eq. (6)), MSE (Eq. (7)), RMSE (Eq. (8)), and MAE (Eq. (9)). These statistical metrics were calculated as follows:

(6) $$R^{2}=1-\frac{\sum_{i=1}^{n}{\left(y_{i}-{\hat{y}}_{i}\right)}^{2}}{\sum_{i=1}^{n}{\left(y_{i}-{\bar{y}}_{i}\right)}^{2}}$$

(7) $$\mathrm{MSE}=\frac{1}{n}\sum_{i=1}^{n}{\left(y_{i}-{\hat{y}}_{i}\right)}^{2}$$

(8) $$\mathrm{RMSE}=\sqrt{\frac{1}{n}\sum_{i=1}^{n}{\left(y_{i}-{\hat{y}}_{i}\right)}^{2}}$$

(9) $$\mathrm{MAE}=\frac{1}{n}\sum_{i=1}^{n}\left|y_{i}-{\hat{y}}_{i}\right|$$

Where $y_{i}$ and ${\hat{y}}_{i}$ are the actual and predicted sensory score of i-th sample, respectively, $\bar{y}\;$is the mean of actual sensory score, and n is the total number of samples.

### 2.4 *E*-nose analysis

PEN3 E-nose (WinMuster Airsense Analytics Inc., Schwerin, Germany) was employed to distinguish aroma compounds using a selective sensor array combined with a modified identification method (Zhu et al., 2020). PEN3 system was composed of 10 metal oxide sensors and sensitive to different compounds (**Table S3**). Briefly, 5 g of solid FCP samples were placed into a 20 mL headspace vessel and stood at room temperature (25 °C) for 30 min to equilibrate before analysis. The e-nose system parameters were set as sample pretreatment time of 5 s, flushing time of 120 s, measurement time of 70 s, and the injection flow rate of 400 mL/min.

### 2.5 Solid-phase microextraction of volatile compounds

1 g of minced sample with saturated NaCl solution (2 mL), 1,2-dichlorobenzene (10 μL, 20 mg/L, internal standard), and a magnetic stirrer were transferred into a 20 mL headspace vial with a lid. After the vial was preheated at 70 °C for 15 min. The SPME fiber (50/30 μm DVB/CAR/PDMS, Sigma-Aldrich, Inc., Bellefonte, PA, USA) was inserted into the headspace vial and extracted at 70 °C for 45 min, and subjected the fiber into the injector port of the GC at 250 °C for 3 min.

### 2.6 GC–MS analysis

GC–MS spectrometry (QP 2010 PLUS, Shimadzu, Japan) combined with a Rtx-5MS capillary column (30.0 m × 0.25 mm × 0.25 μm, Shimadzu Co., Kyoto, Japan) was used for flavor analysis. GC–MS analysis method was modified based on Ma et al. (2022). For GC system: the initial column temperature was maintained at 40 °C for 2 min, then raised to 80 °C at a rate of 4 °C/min and held for 1 min, followed by a second increase to 240 °C at a rate of 3.5 °C/min and held for 4 min. In the split ratio of 1:10 GC inlet mode, high-purity helium (purity>99.999 %) was applied as the carrier gas with a flow rate of 1.0 mL/min. For MS system, ion source temperature was 200 °C and the interface temperature of 220 °C with the solvent delay time of 2 min.

### 2.7 GC × GC-Q-TOF-MS analysis

To comprehensively analyze the volatile compounds of FCP, GC × GC-Q-TOF-MS was used for identification as described by Liu et al. (2024) with some modifications. The GC × GC-Q-TOF-MS set up comprised an Agilent 8890 GC (Agilent, Palo Alto, CA, USA) coupled with an Agilent 7250 GC/Q-TOF-MS (Agilent, Palo Alto, USA). The column set included HP-5 ms Ultra Inert capillary column (15.0 m × 0.25 mm × 0.25 μm, Agilent, USA) as the first dimensional (1D) column and Rxi-17 Sil MS capillary column (21.97 m × 250 μm × 0.25 μm, Agilent, USA) as the second dimensional (2D) column. In the splitless GC inlet mode, high-purity helium (purity>99.999 %) was used as the carrier gas with flow rate of 30.0 mL/min. The temperature program was held at 40 °C for 2 min, and then at 4 °C/min ramping up to 80 °C held for 1 min, followed by a second increased to 240 °C at a rate of 3 °C/min and held for 4 min. The modulation period was set at 7 s, the temperature of ion source and ionization potential of MS were 200 °C and 70 eV, respectively. The temperature of injection port was set at 250 °C.

### 2.8 GC-O analysis

The Agilent 8890 GC equipped with an olfactometry system (ODP 2, Gerstel, Mülheim an der Ruhr, Germany) was used to determine the characteristics of the aroma-active compounds. At the end of the GC capillary column, the isolates were split into a GC/Q-TOF-MS detector and an olfactometry system at a ratio of 1:1. The conditions for GC-O were aligned with GC × GC-Q-TOF-MS, and the sniffing port temperature was held at 250 °C. Two trained evaluators were invited to conduct the GC-O analysis. When the odor appeared in the sniffing port, they were asked to immediately describe the aroma characteristics and aroma intensity according to a 3-point intensity scale from 0 to 3, where “0” represented none, “1” indicated a weak but recognizable aroma, “2” represented medium aroma, and “3” represented strong aroma (Qi et al., 2022).

### 2.9 Qualitative and quantitation analysis

Volatile compounds of FCPs were qualitatively analyzed using mass spectrometry library and linear retention indices (LRIs). Compounds identification was performed by comparing the experimental mass spectra with the NIST 17 mass spectrometry database match degree exceeded 80 % in GC–MS and the similarity and reverse mass spectra>700 and possibility>4000 in GC × GC-Q-TOF-MS. The LRI were analyzed based on the n-alkanes (C_7_-C_40_) under the same GC–MS and GC × GC-Q-TOF-MS detection conditions, and then determined by comparing them with the LRI values of the corresponding target compounds.

Quantitation of the volatile compounds was using external standard curves. Considering the influence of the matrix effect, before the quantitation analysis, the odorless matrix was prepared according to the method as described in the literature Xiao et al. (2024) with slight modifications. A mixture of 50 g FCP and 100 mL ultrapure water was transferred to a rotary evaporation flask and concentrated at 40 °C and 80 rpm under reduced pressure. The process was repeated at least three times until the sample was odorless. Subsequently, the samples were stored in −80 °C refrigerator (DW-HL218, Meiling, Anhui, China), followed by vacuum freeze drying at −40 °C for 24 h using a vacuum freeze dryer (SCIENTZ-10 N, SCIZNTZ, Ningbo, China). The calibration curves were prepared as followed: the mixed aroma compounds standards (0.025 mg/kg to 2.5 mg/kg) were dissolved in anhydrous ethanol and diluted with 10 % (*w*/w) saltwater, then authentic standards (external standard) and 1,2-dichlorobenzene (internal standard) were added to the artificial odorless matrix prepared above. The sample were extracted and identified under the same conditions using gas chromatography-selected ion-monitoring (GC-SIM). The authentic standards and calibration equations were listed in Table 1. Meanwhile, the concentrations of authentic flavor standards were determined in SIM mode, and the aroma compounds in FCP were quantified based on calibration curves.

**Table 1:** Concentration (mean ± standard deviation), aroma description, odor threshold, and OAVs of aroma-active compounds in FCP-1.

Compounds	Aroma description	Concentration(μg/kg)	OT(μg/kg)^a^	OAV	calibration eq^b^	R^2^
3-Methyl-1-butanol		515.65 ± 15.65	220	2	y = 0.0181× + 0.2080	R^2^ = 0.9832
4-Methyl-1-pentanol	sour, stink	125.82 ± 8.96	17	7	y = 0.4077× + 3.9027	R^2^ = 0.9757
Ethyl 2-methylbutyrate	banana, pineapple	79.89 ± 1.77	0.15	533	y = 0.0888× + 0.500	R^2^ = 0.9929
Hexanol		27.70 ± 0.51	5.60	5	y = 0.1387× + 1.0357	R^2^ = 0.9883
Isoamyl acetate		31.84 ± 1.27	2	16	y = 0.1198× + 9.6911	R^2^ = 0.9973
Styrene		37.48 ± 1.44	3.60	10	y = 0.6047× + 0.6777	R^2^ = 0.9875
Methional	soy sauce	1.80 ± 0.80	0.04	45	y = 4.1955× - 2.0162	R^2^ = 0.9742
Ethyl hexanoate		19.54 ± 0.95	2.20	9	y = 1.5617× - 1.4106	R^2^ = 0.9916
Octanal	fatty	14.75 ± 1.18	0.70	21	y = 0.1083× - 0.0386	R^2^ = 0.9938
Hexyl acetate	refreshing	8.69 ± 0.03	2.20	4	y = 2.4288× - 2.4148	R^2^ = 0.9283
β-Limonene		12.69 ± 0.35	10	1	y = 0.3469× + 0.3084	R^2^ = 0.9645
Benzyl alcohol		206.91 ± 82.14	10,000	<1	–	–
Ethyl 2-hexenoate	beer	16.07 ± 0.03	14	1	y = 0.0123× + 0.0916	R^2^ = 0.998
Phenylacetaldehyde	refreshing	59.02 ± 0.79	6.30	9	y = 0.7866× + 0.0632	R^2^ = 0.9589
Trans-Linalool oxide		72.39 ± 7.63	100	<1	–	–
Guaiacol	pungent	5.95 ± 0.91	1.60	4	y = 0.4513× + 3.2355	R^2^ = 0.9934
Linalool	floral	1163.54 ± 37.6	6	194	y = 0.6274× + 2.0115	R^2^ = 0.9792
Nonanal		84.58 ± 35.51	2	42	y = 1.9236× - 0.4719	R^2^ = 0.9348
Phenylethyl Alcohol	rose	1450.79 ± 45.74	60	24	y = 0.2431× + 0.2266	R^2^ = 0.9412
Allocimene B		2.38 ± 0.84	34	<1	–	–
Camphor		4.64 ± 0.48	250	<1	–	–
4-Ethylphenol	stink	269.61 ± 104.35	21	13	y = 1.0118× - 0.3388	R^2^ = 0.9911
3-Isobutyl-2-methoxypyrazine	pepper, green	37.20 ± 2.32	0.038	979	y = 0.1668× + 0.1239	R^2^ = 0.9845
α-Terpineol	clove, woody	525.12 ± 21.32	4.60	114	y = 1.0681× + 4.7893	R^2^ = 0.9879
Methyl salicylate		176.52 ± 7.05	60	3	y = 0.4511× + 0.3847	R^2^ = 0.9906
Ethyl caprylate		97.51 ± 2.78	5	20	y = 0.9715× - 1.0603	R^2^ = 0.9804
2,3-Dihydrobenzofuran		0.83 ± 0.04	3.3	<1	–	–
Phenethyl acetate		47.15 ± 3.09	19	2	y = 15.2100× + 14.7820	R^2^ = 0.9955
Ethyl 2-hydroxybenzoate		186.69 ± 2.67	84	2	y = 2.0685× - 2.2718	R^2^ = 0.9893
Spiroxide	tea-leaf, green	17.22 ± 2.82	0.20	86	y = 0.8631× + 0.2106	R^2^ = 0.9658
4-vinylguaiacol		40.25 ± 5.77	19	2	y = 11.5390× - 5.5579	R^2^ = 0.9184
Ethyl trans −4-decenoate	fruity	1549.30 ± 40.67	112	14	y = 2.4522× - 2.6083	R^2^ = 0.9902
Ethyl isovalerate		20.59 ± 5.15	0.20	103	y = 3.8334× - 1.9437	R^2^ = 0.9011
Ethyl 3-phenylpropionate	seasoning	20.47 ± 1.13	100	<1	–	–
γ-Nonanolactone		7.82 ± 2.69	66	<1	–	–
β-Damascenone	sweaty, honey	26.92 ± 0.58	0.10	269	y = 14.8510× + 3.6142	R^2^ = 0.9935
Ethyl cinnamate		1.84 ± 0.34	-^c^	–	–	–
β-Ionone	fruity, floral	232.60 ± 221.00	0.40	582	y = 4.3626× - 1.7293	R^2^ = 0.9826
Nerolidol	fruity	442.62 ± 3.55	10	44	y = 5.6797× - 2.2518	R^2^ = 0.9994
Ethyl laurate		404.22 ± 4.35	400	1	y = 0.8717× + 0.2306	R^2^ = 0.9857
Hexyl 2-methylbutyrate		29.09 ± 5.88	3	10	y = 0.9863× - 0.4788	R^2^ = 0.9291
Ethyl caprate		47.01 ± 13.75	5	10	y = 0.0196× + 0.2441	R^2^ = 0.8622

a, Odor thresholds in water taken from the literature. b, Equations of standard curves. c, The odor threshold was not queried from the literature.

### 2.10 OAV analysis

OAV was used to assess the contribution of volatile compounds. The OAV was calculated using Eq. (10):

(10) $$\mathrm{OAV}=\frac{c}{\mathrm{OT}}$$

Where, c is the concentration of the volatile compounds, and OT is the odor threshold of the compounds.

### 2.11 Aroma recombination and omission experiments

Thirty-four aroma compounds (OAVs≥1) in FCP-1 sample was selected for aroma recombination experiments. They were mixed and added to the artificial odorless matrix according to their actual concentration in the sample, then the vial was covered and stood for half an hour. The panelists evaluated the similarity between the FCP-1 sample and recombinant model. The aroma omission experiments were conducted by individually omitting a single aroma compound from the comprehensive recombinant model, and then the panelists evaluated the difference between the omission models and the recombinant model to determine the key aroma compounds in a triangle test.

### 2.12 Sensory evaluation

The sensory evaluation consisted of 9 evaluators (4 males and 5 females, aged 22–28), who were recruited from the School of Food Science and Technology at Hunan Agricultural University. Each panelist had received professional training and had at least 2 years of experience in the sensory evaluation of chili-based products. All the evaluators were trained according to the method in the literature (Wang et al., 2020). The training process was as follows: each panelist received up to 90 h of sensory training to become familiar with the sensory descriptors and aroma intensity of FCP. This training enhanced their ability to accurately recognize and assess key aroma characteristics. Aroma standards applied in the training were categorized into some groups according to the aroma characteristics: linalool as floral odors, hexanal as green, 2-methoxy-3-isobutyl pyrazine as pepper, isobutyl acetate as fruity, soy sauce as soy sauce-like aroma, 4-methyl-1-pentanol as sour stink and fermented aroma, β-damascenone as sweety, α-terpineol as woody, and heptaldehyde as fatty. Sensory analysis was conducted in an odor-free room at 25 ± 1 °C. FCP sample (10 g) was placed in an unflavored plastic cup for evaluation. Then, panelists recorded sensory descriptors and rated the intensity of each attribute on a 5-point scale ranging from 0 (none) to 5 (very strong) (Cong et al., 2023). Additionally, they were asked to rest between evaluating different samples to relieve olfactory fatigue.

### 2.13 Statistical analysis

Significance analysis was used by SPSS 26.0 (IBM, USA). Radar charts, 3D surface plots and Venn graphs were created using Origin 2021 software (Northampton, USA). Scatterplots and Stacked bar graphs were drawn by GraphPad Prism 8 (GraphPad Software, USA). MATLAB R2016b software (MathWorks, USA) was used to construct the models and training performance plot was automatically generated. Linear discriminant analysis (LDA) was performed using the instrument's built-in software.

## 3 Results and discussions

### 3.1 Models screening for predicting sensory scores of FCP

**Fig. 1:**
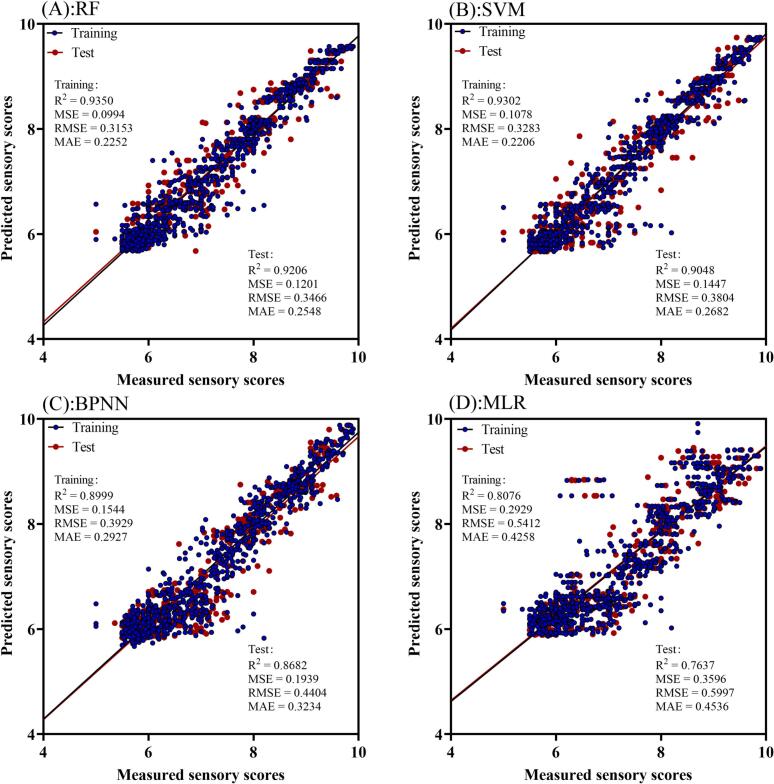
Regression plots of predicted vs. measured sensory scores for training and test sets using RF (A), SVM (B), BPNN (C), and MLR (D) models.

RF is an ensemble algorithm that aggregated multiple decision trees, demonstrated strong performance in capturing nonlinear interactions and mitigating overfitting through bootstrap aggregation. Its robustness to noise and suitability for high-dimensional data make it particularly well-suited for processing e-nose datasets. After grid search optimization, the best performance was achieved using 150 trees and 10-fold cross-validation (**Fig.S2A)**, The model yielded high predictive accuracy with R^2^ of 0.9350 and 0.9206, and low MSE of 0.0994 and 0.1201, RMSE of 0.3153 and 0.3466, and MAE of 0.2252 and 0.2548 on the training and test sets, respectively (Fig. 1A). These results confirmed that RF is well-suited for sensory prediction, offering a good balance between accuracy and stability. Nevertheless, its ensemble structure limits interpretability, making it less suitable for understanding individual variable contributions.

SVM selecting an appropriate kernel function and optimizing its parameters are critical steps for model performance. Among various kernel functions, the Radial Basis Function (RBF) was selected for its strong ability to model nonlinear patterns between e-nose sensor responses and human sensory evaluations (Dong et al., 2014). Through a grid search with k-fold cross-validation, the optimal hyperparameters were set as C = 10 and γ = 1 (**Fig. S2B**). As shown in Fig. 1B, the SVM model achieved R^2^ of 0.9302 and 0.9048, MSE of 0.1078 and 0.1447, RMSE of 0.3283 and 0.3804, and MAE of 0.2206 and 0.2682 on the training and test set, respectively. These results demonstrate the model's capability in modeling complex sensory relationships. However, SVM performance was found to be highly sensitive to hyperparameter tuning, which increases computational cost and reduces practical flexibility.

To leverage the flexibility of BPNN in capturing complex sensory perception patterns, we systematically optimized the number of hidden layers, neurons, and training epochs. Underfitting can occur with insufficient hidden units, while excessive complexity may lead to overfitting. After testing various configurations, the optimal architecture was identified as comprising 3 hidden layers with 25 neurons each and 19 training epochs (**Fig.S2C**). The model achieved R^2^ of 0.8999 and 0.8682, MSE of 0.1544 and 0.1939, RMSE of 0.3929 and 0.4404, and MAE of 0.2927 and 0.3234, on the training and test set, respectively (Fig. 1C). While BPNN captured complex nonlinearities, it was highly sensitive to overfitting and required careful architecture tuning, especially for small sample sizes.

MLR served as a baseline model to assess the potential of linear relationships in the dataset. Although MLR offered simplicity and high interpretability, its predictive power was limited, achieved R^2^ of only 0.8076 and 0.7637, MSE of 0.2929 and 0.3596, RMSE of 0.5412 and 0.5997, and MAE of 0.4258 and 0.4536 on the training and test set, respectively, (Fig. 1D). The relatively poor performance indicated that linear models could not sufficiently capture the complexity of sensory perception based on volatile profiles.

In summary, among all evaluated models, RF outperformed others in terms of predictive accuracy and generalization, while maintaining low sensitivity to parameter tuning. SVM also performed well but was more computationally intensive. BPNN showed strong potential for complex pattern recognition but required careful design to avoid overfitting. MLR, though interpretable, was inadequate for capturing the nonlinear nature of the dataset. These comparisons highlighted the advantages of nonlinear ensemble methods, particularly RF, in modeling sensory attributes of FCPs from e-nose data.

### 3.2 Prediction of sensory scores for eight types of FCPs

**Fig. 2:**
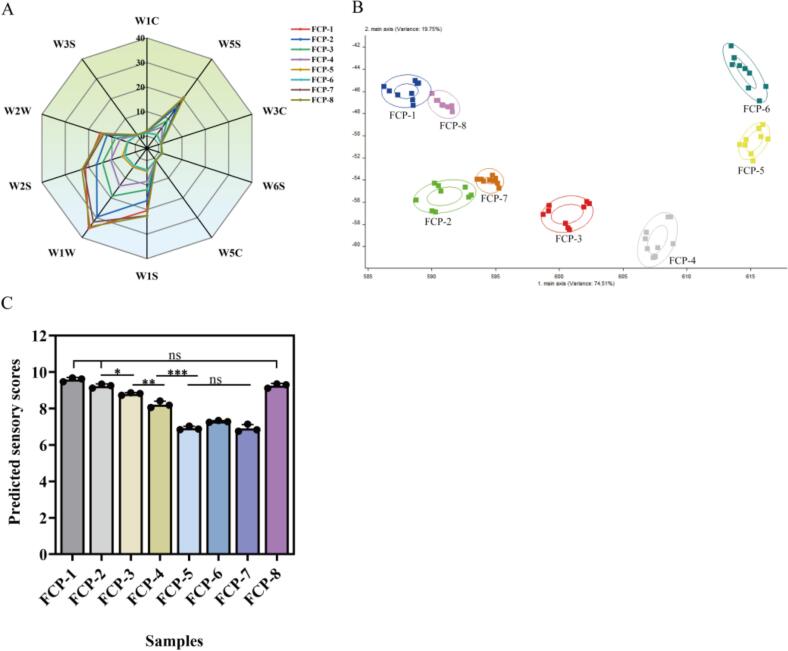
Radar chart of e-nose sensor responses for 8 types of fermented chopped peppers **(A)**, LDA analysis results **(B)**, and Bar chart of predicted sensory scores in the eight types of ferment chopped peppers **(C).**

*E*-nose analysis found that significant differences in response intensities of certain sensors among FCP samples from different regions, particularly on W5S, W1S, W1W, W2S, and W2W, as shown in Fig. 2A. These sensors were known to respond to compounds commonly associated with pepper, fermented, fruity, and soy sauce-like aroma (Feng et al., 2022; Li, Tian, et al., 2022). The differences in signal intensity illustrated that the concentration and composition of aroma-active compounds varied across samples, potentially due to differences in raw materials or microbial communities. In addition, LDA was analyzed to visualize the distribution pattern of FCP samples from different regions based on e-nose (Zhu et al., 2020). LD1 and LD2 contributed 74.51 % and 19.75 %, respectively, effectively capturing the major differences in volatile profiles (Fig. 2B**)**. The eight samples were clearly separated, suggesting distinct aroma profiles, while the FCP-1 and FCP-8 were close, indicating potential similarity in their aroma composition.

Based on the e-nose data, the trained RF model was applied to predict the sensory scores of the FCP samples, as shown in Fig. 2C, the predicted sensory score ranking was: FCP-1 > FCP-8 > FCP-2 > FCP-3 > FCP-4 > FCP-6 > FCP-7 > FCP-5. Among them, FCP-1 and FCP-8 showed the highest predicted sensory scores, which aligned with their proximity in LDA. They may certain favorable volatile compounds (such as higher levels of esters, alcohols, and sulfur compounds) that contributed positively to aroma perception and consumer acceptance The ranking was consistent with the LDA results further validating the robustness of the RF model, which accurately identified difference in sensor responses and showed strong potential in predicting sensory performance and distinguishing aroma characteristics across samples.

### 3.3 Determination of volatile compounds by GC–MS

GC–MS was used to characterize volatile compounds in eight types of FCPs. A total of 53 volatile compounds were identified, including 1 acid, 4 ketones, 12 alcohols, 19 esters, 7 aldehydes, 1 pyrazine and 8 alkanes (**Table S4**). As shown in **Fig.S3 A**, FCP-1 contained the largest number of volatile compounds (38), while FCP-5 presented the fewest (14). Correspondingly, **Fig.S3B** illustrated that FCP-1 exhibited the highest total volatile concentration (1855.4 μg/kg), while FCP-5 had the lowest (267.7 μg/kg). These results were consistent with the sensory score predictions based on the e-nose and RF model in section 3.2**,** where FCP-1 and FCP-5 received the highest and lowest predicted scores, respectively. The higher abundance and diversity of volatile compounds in FCP-1 may contribute to its superior aroma profile and favorable sensory perception. The observed differences in volatile compounds were likely attributed to variations in raw materials, which played a crucial role in shaping the final aroma characteristics. This finding aligned with previous found that pepper varieties and regional origin significantly impacted the volatile characteristics of FCP products (Ye et al., 2022).

### 3.4 Determination of volatile compounds by GC × GC-O-Q-TOF-MS

**Fig. 3:**
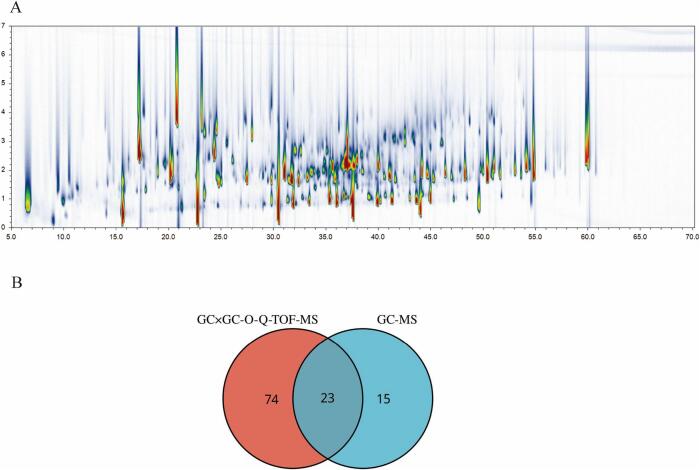
GC × GC-O-Q-TOF-MS analysis of representative topographic maps of volatile compounds **(A)**, Venn graph of GC × GC-O-Q-TOF-MS and GC–MS volatile compounds **(B)** in FCP-1.

To further investigate the aroma composition of the top-performing sample (FCP-1), volatile compounds profiling was carried out using GC × GC-O-Q-TOF-MS (a more accurate technique). The representative topographic maps obtained through GC × GC-O-Q-TOF-MS analysis were shown in Fig. 3A. A total of 97 volatile compounds (16 alcohols, 42 esters, 9 aldehydes, 5 phenols, 1 pyrazine, 5 ketones, 1 acid, 3 furans, 15 alkenes, and with 19 of them identified as odor active) were detected in FCP-1 (**Table S5**). As shown in Fig. 3B, 74 compounds, such as ethyl hex-2-enoate, 4-vinylguaiacol, β-damascenone, β-ionone, and others, were only detected in the GC × GC-O-Q-TOF-MS. And some compounds like nonanal, and ethyl caprate, etc., were not in GC × GC-O-Q-TOF-MS (these compounds were only detected by GC–MS). These findings suggested that the combination of these two methods can provide a more comprehensive understanding of the volatile compounds in FCP-1.

Alcohols were generated through sugar metabolism, as well as from the decarboxylation and dehydrogenation of amino acids. In FCP-1, five alcohols were identified by GC-O, among which phenylethyl alcohol (1450.79 ± 45.74 μg/kg) was the most abundant, followed by linalool, 4-methyl-1-hexanol, α-terpineol, and nerolidol. Linalool and phenylethyl alcohol were known for the “floral” notes and were closely associated with the microbial fermentation of glycosides (Wang et al., 2021). Phenylethyl alcohol could be formed through two pathways: benzene acetaldehyde/phenethylamine route and trans-cinnamic acid/phenyl lactate (Gao et al., 2021). 4-Methyl-1-pentanol (“sour” and “stink” aroma) might be a product of yeast fermentation during pepper fermentation (Xiao et al., 2024), while α-terpineol (“clover” and “woody” aroma) might be associated with the addition of spices (Gao et al., 2024). Nerolidol was a terpenoid alcohol that imparted a “fruity” aroma. And these compounds had demonstrated some renal protective effects (Hammad et al., 2023).

For aldehyde compounds, GC-O analysis detected phenylacetaldehyde, methional and octanal. Phenylacetaldehyde was associated with “refreshing” aroma. Octanal imparted a “fatty” aroma. Methional was the only organic sulfur compound, possessing a characteristic “soy sauce” aroma (Lulu et al., 2020). Esters were generated through the process of esterification, where acids and alcohols combined to impart fruity and floral aromas to the samples, and were often considered as a signal of maturity in fermented foods (Yu et al., 2019). A total of five esters were determined using GC-O in FCP-1. Wherein, ethyl (4E)-4-decenoate (“fruity” and “soy sauce” aroma) was recognized as a crucial volatile compound that added depth and richness to the FCP. Other esters detected included ethyl 2-methylbutyrate, hexyl acetate, ethyl hex-2-enoate, ethyl 3-phenylpropionate contributed fruity, beer-like, and seasoning aroma, enhancing the sensory complexity of FCP-1.

Ketones could be generated through the degradation of amino acid or microbial oxidation. β-ionone had a floral and fruity aroma, which was derived from the oxidative degradation of β-carotene during the curing process of pepper (Wang et al., 2020). β-damascenone (sweaty and honey) was also detected. Noticeably, for the first time, camphor was detected in FCP and exhibited a cucumber odor. Phenols might be the secondary metabolites of aromatic compounds produced through microbial enzymatic hydrolysis during pepper fermentation (Guo et al., 2022). Wherein, guaiacol (“pungent” aroma) and 4-ethylphenol (“stink” aroma) detected in the sample has been identified as a contributor to the characteristic aroma of certain fermented foods, like Japanese miso (Kumazawa et al., 2013). In this case, phenols might play an important role in aroma formation of FCP product. Furthermore, 2-methoxy-3-isobutyl pyrazine and spiroxide were detected in FCP-1. 3-isobutyl-2-methoxypyrazine, known for its “peppery” and “green” aroma, was likely generated from lipid oxidation and amino groups (Wu et al., 2024). Spiroxide was a rare monoterpene compound that had a “tea-leaf” and “green” aroma, primarily found in tea.

### 3.5 Identification of key aroma compounds through OAV

To identify the key aroma compounds in FCP-1, OAV was used to assess the contribution of individual volatile compounds to the overall aroma. Typically, compounds with OAVs≥1 were initially considered the key aroma contributors to FCP-1's overall aroma (Liu et al., 2019). In this study, a total of 34 volatile compounds in FCP-1 were identified by OAVs≥1, including 7 alcohols, 14 esters, 1 pyrazine, 2 ketones, 4 aldehydes, 3 phenols, and 3 alkenes (Table 1). 3-isobutyl-2-methoxypyrazine exhibited the highest OAV (OAV = 979), strongly influencing the characteristic “pepper” aroma profile of the FCP. 6 volatile compounds with OAV > 100, such as ethyl 2-methylbutyrate (OAV = 533), linalool (OAV = 194), α-terpineol (OAV = 114), β-damascenone (OAV = 269), β-ionone (OAV = 582), and ethyl isovalerate (OAV = 103), also contributed strongly to the overall aroma profile. These high OAV values indicated that these compounds played a prominent role in enhancing the complexity and richness of FCP-1's aroma. Compound with OAVs between 10 and 100, such as isoamyl acetate (OAV = 16), styrene (OAV = 10), methional (OAV = 45), octanal (OAV = 21), nonanal (OAV = 42), phenethyl alcohol (OAV = 24), 4-ethylphenol (OAV = 13), ethyl caprylate (OAV = 20), spiroxide (OAV = 86), ethyl (4E)-4-decenoate (OAV = 14), nerolidol (OAV = 44) and ethyl caprate (OAV = 20) were considered moderate contributors to the aroma profile. Finally, 16 compounds had relatively low OAV value (range from 1 to 10), which were 3-methyl-1-butanol, 4-methyl-1-pentanol, hexanol, ethyl isohexanoate, hexyl 2-methylbutyrate, ethyl caprate, hexyl acetate, β-limonene, ethyl 2-hexenoate, phenylacetaldehyde, guaiacol, methyl salicylate, phenethyl acetate, ethyl 2-hydroxybenzoate, 4-vinylguaiacol, and ethyl laurate, indicating that they might have a mild impact on the overall aroma. Although these compounds may not be as dominant, these compounds still contribute to the overall sensory perception.

### 3.6 Aroma recombination analysis

**Fig. 4:**
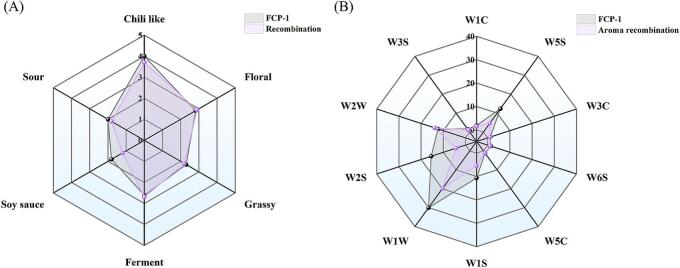
Radargram of Personnel sensory **(A)** or E-nose Sensory **(B)**.

To further confirm the contribution of compounds with OAVs≥1 to the overall aroma of FCP-1, recombination experiments were conducted. The recombination experiment model was prepared based on odorless matrix, 10 % (*w*/w) saltwater, and volatile compounds with OAV ≥ 1. Then evaluated by nine trained panelists to assess its similarity to the aroma profile of FCP-1. Six sensory properties, including “soy sauce”, “ferment”, “sour”, “chili-like”, “grassy”, and “floral” were chosen to be the main characteristics. The recombination model was highly similar to the sample FCP-1 in terms of “sour”, “chili-like”, “ferment”, “grassy” and “floral” aromas as shown in Fig. 4A. However, the recombination model had slightly weak “soy sauce” attribute. Compounds with “soy sauce” aroma was also not fully simulated in Douchi, as similarly reported by Wu et al. (2024). This discrepancy may be due to incomplete reconstitution of all aroma compounds, particularly those with complex sensory profile associated with amino acid and fermentation byproducts contributing to “soy sauce” characteristics, as well as the lack of furanone (soy sauce, metallic) or some unknown compounds, which require further investigation (Steinhaus & Schieberle, 2007).

As shown in Fig. 4B, the e-nose sensory analysis demonstrated that the FCP-1 sample and the recombination model were sensitive to the same e-nose sensors, particularly the W5S, W1S, W1W, W2S, and W2W sensors. However, several sensors showed significantly higher responses in the FCP-1 sample than the recombination model, which may be attributed to matrix effects influencing the release and perception of aroma compounds (Wang et al., 2020; Xiao et al., 2024). Firstly, the detected odor thresholds may not fully reflect those in a real food matrix. Furthermore, accurately capturing the complex interactions between odorants and the matrix components remained challenging. For example, the 10 % (w/w) saltwater medium may alter the volatility and release of aroma compounds by altering their solubility and interactions, thereby reducing their detection by e-nose. This may partly explain the reduced responses of sensors such as W5S sensor (sensitive to nitrogenous compounds) and W1S sensor (sensitive to alkanes). Additionally, the simplified matrix in the recombination sample would lack the complex interactions found in a real food matrix (Xiao et al., 2024), where the food's amino acids, organic acids, capsaicin, proteins, and other components interacted with volatiles to influence their release dynamics. These missing interactions might also lead to decreased retention or altered release patterns of the volatiles detected by W5S, W1S, W1W, W2S, and W2W sensors.

### 3.7 Aroma omission analysis

A total of 34 omission models were prepared for the triangle tests to further investigate the contribution of specific compounds to the overall aroma in FCP-1. Each omission model was generated by systematically removing one of the 34 volatile compounds with OAVs≥1 to assess its individual contribution. Triangle tests were then exploited to compare the recombination models with the omission models. According to the results shown in Table 2, linalool, phenethyl alcohol, methional, 2-methoxy-3-isobutyl pyrazine, ethyl trans-4-decenoate, β-ionone, and spiroxide had the highest differences (*p* < 0.01), and thus have the greatest contribution to the overall aroma of FCP-1. The lack of ethyl 2-methylbutyrate, α-terpineol, 4-ethylphenol, β-damascenone, and nerolidol also resulted in significant differences (*p* < 0.05), showing that these five odorants had an essential contribution to the overall aroma. However, the omission of 4-methyl-1-pentanol, isoamyl acetate, styrene, ethyl hexanoate, hexyl acetate, octanal, ethyl 2-hexenoate, phenylacetaldehyde, methyl salicylate, ethyl caprylate, hexyl 2-methylbutyrate, phenethyl acetate, ethyl 2-hydroxybenzoate, 4-vinylguaiacol, ethyl laurate, hexanol, ethyl caprate, nonanal, 3-methyl-1-butanol, limonene, guaiacol, and ethyl isovalerate did not lead to a perceivable difference in aroma. The results of omission experiments were mostly consistent with the compounds' higher OAVs, except for nonanal (OAV = 42) and octanal (OAV = 21). This may be attributed to the presence of other compounds with similar sensory characteristics that compensated for their absence, thereby minimizing their perceptual impact.

**Table 2:** Omission experiments from the recombinant model.

Aroma-active compounds omitted from the recombinant model	FCP-1	
	N^a^	Significance^b^
Linalool	9	**
4-Methyl-1-pentanol	3	
Phenethyl alcohol	7	**
Ethyl 2-methylbutyrate	6	*
3-Isobutyl-2-methoxypyrazine	9	**
Isoamyl acetate	4	
Ethyl trans-4-decenoate	7	**
Styrene	5	
Methional	9	**
Ethyl Hexanoate	3	
Hexyl acetate	2	
Octanal	5	
Ethyl 2-hexenoate	4	
Phenylacetaldehyde	5	
4-Ethylphenol	6	*
α-Terpineol	6	*
Methyl salicylate	5	
Ethyl caprylate	5	
Hexyl 2-methylbutyrate	4	
Phenethyl acetate	2	
Ethyl 2-hydroxybenzoate	5	
4-Vinylguaiacol	5	
β-Damascenone	6	*
β-Ionone	7	**
Ethyl laurate	4	
Nerolidol	6	*
Hexanol	4	
Ethyl caprate	5	
Nonanal	4	
3-Methyl-1-butanol	5	
Limonene	1	
Guaiacol	3	
Ethyl isovalerate	2	
Spiroxide	8	**

a The number of correct judgements by 9 panelists after the evaluation of aroma differences by the triangle test. ^b^ levels of significance: * significant (*p* ≤ 0.05); **, highly significant (*p* ≤ 0.01).

## 4 Conclusion

The RF, SVM, MLR, and BPNN models were optimized and used to predict sensory scores of FCPs based on e-nose data, RF model with 10-fold cross-validation and 150 trees achieved the highest accuracy in the four models. GC–MS, *E*-nose and the trained RF model were applied to analysis the volatile compounds and predict the sensory scores for eight types of FCPs. And then, 19 kinds of aroma-active compounds were separated by GC-O in the top-performing sample (FCP-1), 97 of which were identified by GC × GC-O-Q-TOF-MS. Among 34 odorants (OAV ≥ 1) were recognized as important aroma compounds. Further aroma recombination and omission experiments verified that the recombinant model composed of these compounds had a certain similarity to the real sample; linalool, phenethyl alcohol, methional, 3-isobutyl-2-methoxypyrazine, ethyl trans-4-decenoate, β-ionone, spiroxide, ethyl 2-methylbutyrate, α-terpineol, 4-ethylphenol, β-damascenone, and nerolidol were the key aroma compounds in FCP-1. This study provided the novel method for predicting the sensory score and the useful information for understanding the aroma composition in FCP-1 sample.

## CRediT authorship contribution statement

**Yuan Liu:** Writing – review & editing, Writing – original draft, Methodology, Investigation. **Lingyan Zhao:** Methodology, Funding acquisition. **Chunya Yang:** Validation, Supervision. **Yeyou Qin:** Resources. **Li Zhu:** Writing – review & editing, Supervision. **Fangming Deng:** Funding acquisition, Conceptualization.

## Ethical statement

This study involved sensory analyses conducted by a trained panel and adhered to the principles outlined in the 1964 Helsinki Declaration and its subsequent amendments. The nature of the study was low-risk, involving sensory evaluation of food products, and thus did not require formal approval from an ethics committee. However, the research was conducted in full compliance with ethical principles to ensure the protection of participants' rights and welfare. Informed consent was obtained from all participants involved in the study. They were explicitly informed of their right to decline participation or withdraw their consent at any stage without facing any negative consequences. To ensure confidentiality, all data were anonymized and stored securely. While no formal ethics committee was available, the research followed the principles and guidelines set forth in the Helsinki Declaration to ensure the ethical treatment of participants.

## Declaration of competing interest

The authors declare that they have no known competing financial interests or personal relationships that could have appeared to influence the work reported in this paper.

## Data Availability

The authors do not have permission to share data.

## Appendix A Supplementary data

Supplementary material: Figure. S1, Figure. S2, Figure. S3, Table. S1, Table. S2, Table. S3, Table. S4, Table. S5 Image 1

## References

[bb0005] Cong L., Yuyu Z., Ping Z., Peng W., Honglei T. (2023). Characterization of the key aroma compounds in four varieties of pomegranate juice by gas chromatography-mass spectrometry, gas chromatography-olfactometry, odor activity value, aroma recombination, and omission tests. Food Science and Human Wellness.

[bb0010] Dong J., Li Q., Yin H., Zhong C., Hao J., Yang P., Tian Y., Jia S.R. (2014). Predictive analysis of beer quality by correlating sensory evaluation with higher alcohol and ester production using multivariate statistics methods. Food Chemistry.

[bb0015] Feng X., Wang H., Wang Z., Huang P., Kan J. (2022). Discrimination and characterization of the volatile organic compounds in eight kinds of huajiao with geographical indication of China using electronic nose, HS-GC-IMS and HS-SPME-GC–MS. Food Chemistry.

[bb0020] Gao X., Yu M., Han X., Song H., Pan W., Chen W., Xiong W. (2024). Characterization of odor-active compounds in Liuzhou River snail rice noodles soup by sensory-directed flavor analysis. Journal of Food Composition and Analysis.

[bb0025] Gao X.L., Feng T., Sheng M., Wang B., Wang Z., Shan P., Ma H. (2021). Characterization of the aroma-active compounds in black soybean sauce, a distinctive soy sauce. Food Chemistry.

[bb0030] Guan S.H., Liu C.X., Yao Z.P., Wan H.J., Ruan M.Y., Wang R.R., Cheng Y. (2024). Detection and analysis of VOCs in cherry tomato based on GC-MS and GC× GC-TOF MS techniques. Foods.

[bb0035] Guo Q., Adelina N.M., Hu J., Zhang L., Zhao Y. (2022). Comparative analysis of volatile profiles in four pine-mushrooms using HS-SPME/GC-MS and E-nose. Food Control.

[bb0040] Hammad F.T., Al-Salam S., Ahmad R., Yasin J., Hammad A.F., Rasheed J.A., Lubbad L. (2023). The Effect of Nerolidol Renal Dysfunction following Ischemia–Reperfusion Injury in the Rat. Nutrients.

[bb0045] Huang X., Chen T., Zhou P.Y., Huang X.X., Liu D., Jin W.X., Gao Z.H. (2022). Prediction and optimization of fruit quality of peach based on artificial neural network. Journal of Food Composition and Analysis.

[bb0050] Jiang S., Zhu Y., Peng J., Zhang Y., Zhang W., Liu Y. (2023). Characterization of stewed beef by sensory evaluation and multiple intelligent sensory technologies combined with chemometrics methods. Food Chemistry.

[bb0055] Kumazawa K., Kaneko S., Nishimura O. (2013). Identification and characterization of volatile components causing the characteristic flavor in miso (Japanese fermented soybean paste) and heat-processed miso products. Journal of Agricultural and Food Chemistry.

[bb0060] Li S., Tian Y., Sun M., Liu J., Bai Y., Liu X., Guo Y. (2022). Characterization of key aroma compounds in fermented bamboo shoots using gas chromatography-olfactometry-mass spectrometry, odor activity values, and aroma recombination experiments. Foods.

[bb0065] Li X., Cheng X., Yang J., Wang X., Lü X. (2022). Unraveling the difference in physicochemical properties, sensory, and volatile profiles of dry chili sauce and traditional fresh dry chili sauce fermented by lactobacillus plantarum PC8 using electronic nose and HS-SPME-GC-MS. Food Bioscience.

[bb0070] Liu H., Wang Z., Zhang D., Shen Q., Pan T., Hui T., Ma J. (2019). Characterization of key aroma compounds in Beijing roasted duck by gas chromatography–olfactometry–mass spectrometry, odor-activity values, and aroma-recombination experiments. Journal of Agricultural and Food Chemistry.

[bb0075] Liu M., Deng N., Li H., Hou X., Zhang B., Wang J. (2024). Characterization and comparison of flavors in fresh and aged fermented peppers: Impact of different varieties. Food Research International.

[bb0080] Lulu W., Shanshan F., Yan Y., Liang Y., Shuang C., Yan X. (2020). Characterization of potent odorants causing a pickle-like off-odor in Moutai-aroma type baijiu by comparative aroma extract dilution analysis, quantitative measurements, aroma addition, and omission studies. Journal of Agricultural and Food Chemistry.

[bb0085] Ma D., Li Y., Chen C., Fan S., Zhou Y., Deng F., Zhao L. (2022). Microbial succession and its correlation with the dynamics of volatile compounds involved in fermented minced peppers. Frontiers in Nutrition.

[bb0090] Omatu S., Yano M. (2016). E-nose system by using neural networks. Neurocomputing.

[bb0095] Prescott J. (2017). Some considerations in the measurement of emotions in sensory and consumer research. Food Quality and Preference.

[bb0100] Qi S., Wang P., Zhan P., Tian H. (2022). Characterization of key aroma compounds in stewed mutton (goat meat) added with thyme (Thymus vulgaris L.) based on the combination of instrumental analysis and sensory verification. Food Chemistry.

[bb0105] Steinhaus P., Schieberle P. (2007). Characterization of the key aroma compounds in soy sauce using approaches of molecular sensory science. Journal of Agricultural and Food Chemistry.

[bb0110] Wang M.Q., Ma W.J., Shi J., Zhu Y., Lin Z., Lv H.P. (2020). Characterization of the key aroma compounds in Longjing tea using stir bar sorptive extraction (SBSE) combined with gas chromatography-mass spectrometry (GC–MS), gas chromatography-olfactometry (GC-O), odor activity value (OAV), and aroma recombination. Food Research International.

[bb0115] Wang R., Sun J., Lassabliere B., Yu B., Liu S.Q. (2021). Β-Glucosidase activity of Cyberlindnera (Williopsis) saturnus var. mrakii NCYC 2251 and its fermentation effect on green tea aroma compounds. LWT.

[bb0120] Wijaya D.R., Handayani R., Fahrudin T., Kusuma G.P., Afianti F. (2024). Electronic nose and optimized machine learning algorithms for noninfused aroma-based quality identification of Gambung green tea. IEEE Sensors Journal.

[bb0125] Wu Z., Chao J., Tang H., Liu T., Jiang L., Liu Y. (2024). Characterization of key aroma-active compounds in different types of Douchi based on molecular sensory science approaches. Food Chemistry: X.

[bb0130] Xiao, Y., Zhang, S., Wang, X., Zhao, X., Liu, Z., Chu, C., Wang, Y., Hu, X., & Yi, J. (2024). Characterization of key aroma-active compounds in fermented chili pepper (Capsicum frutescens L.) using instrumental and sensory techniques. Food Chemistry*: X*, 101581. doi:10.1016/j.fochx.2024.101581. PMC11260950 39040151

[bb0135] Xu X., Wu B., Zhao W., Lao F., Chen F., Liao X., Wu J. (2021). Shifts in autochthonous microbial diversity and volatile metabolites during the fermentation of chili pepper (Capsicum frutescens L.). Food Chemistry.

[bb0140] Yan J., Zhang A., Sun R., Zhang C., Wang X., Li Z., Yin Y., Liu T. (2025). Aroma evaluation for chili pepper using an E-nose combined with a novel feature fusion technology based on machine learning. Journal of Food Composition and Analysis.

[bb0145] Yang Y.H., Zhao J., Du Z.Z. (2022). Unravelling the key aroma compounds in the characteristic fragrance of Dendrobium officinale flowers for potential industrial application. Phytochemistry.

[bb0150] Ye Z., Shang Z., Li M., Zhang X., Ren H., Hu X., Yi J. (2022). Effect of ripening and variety on the physiochemical quality and flavor of fermented Chinese chili pepper (Paojiao). Food Chemistry.

[bb0155] Yu H., Xie T., Xie J., Ai L., Tian H. (2019). Characterization of key aroma compounds in Chinese rice wine using gas chromatography-mass spectrometry and gas chromatography-olfactometry. Food Chemistry.

[bb0160] Zhang Q., Tang J., Deng J., Cai Z., Jiang X., Zhu C. (2024). Effect of capsaicin stress on aroma-producing properties of lactobacillus plantarum CL-01 based on E-nose and GC–IMS. Molecules.

[bb0165] Zhu D., Ren X., Wei L., Cao X., Ge Y., Liu H., Li J. (2020). Collaborative analysis on difference of apple fruits flavour using electronic nose and electronic tongue. Scientia Horticulturae.

[bb0170] Zou M., Tang H., Chen X., Guo L., Lin J. (2023). Insights into volatile flavor compound variations and characteristic fingerprints in Longpai soy sauce moromi fermentation via HS-GC-IMS and HS-SPME-GC× GC-ToF-MS. LWT.

