# Supplementary material for: Sensory score prediction and key aroma compounds characterization in fermented chopped pepper
Source: Food Chem X. 2025 Jul 5;29:102743. doi: 10.1016/j.fochx.2025.102743 (PMC12272893; doi:10.1016/j.fochx.2025.102743)
Supplement: Supplementary file 1 — Supplementary material: Figure. S1, Figure. S2, Figure. S3, Table. S1, Table. S2, Table. S3, Table. S4, Table. S5 [file mmc1.docx]

**Supplement Materials**

Figure caption

Figure. S1. E-nose and panel sensory score dataset for sensory prediction model construction

Figure. S2. 3D surface plots of parameter optimization for Random Forest (A) and Support Vector Machine (B), and training performance plot for the back propagation neural network model (C).

Figure. S3. Stacked bar graph of volatile compounds Number (A), and Concentration (B) in the eight types of ferment chopped pepper.

Table caption

Table S1 The eight samples of fermented chopped peppers from different regions.

Table S2 Varieties and regions of fermented chopped pepper samples

Table S3 Sensors used in PEN3 e-nose and their performance description

Table S4 Volatile compounds concentration (mean ± standard deviation) in eight types of fermented chopped pepper using GC-MS.

Table S5 Volatile compounds concentration (mean ± standard deviation) in FCP-1 using GC×GC-O-Q-TOF-MS.

| 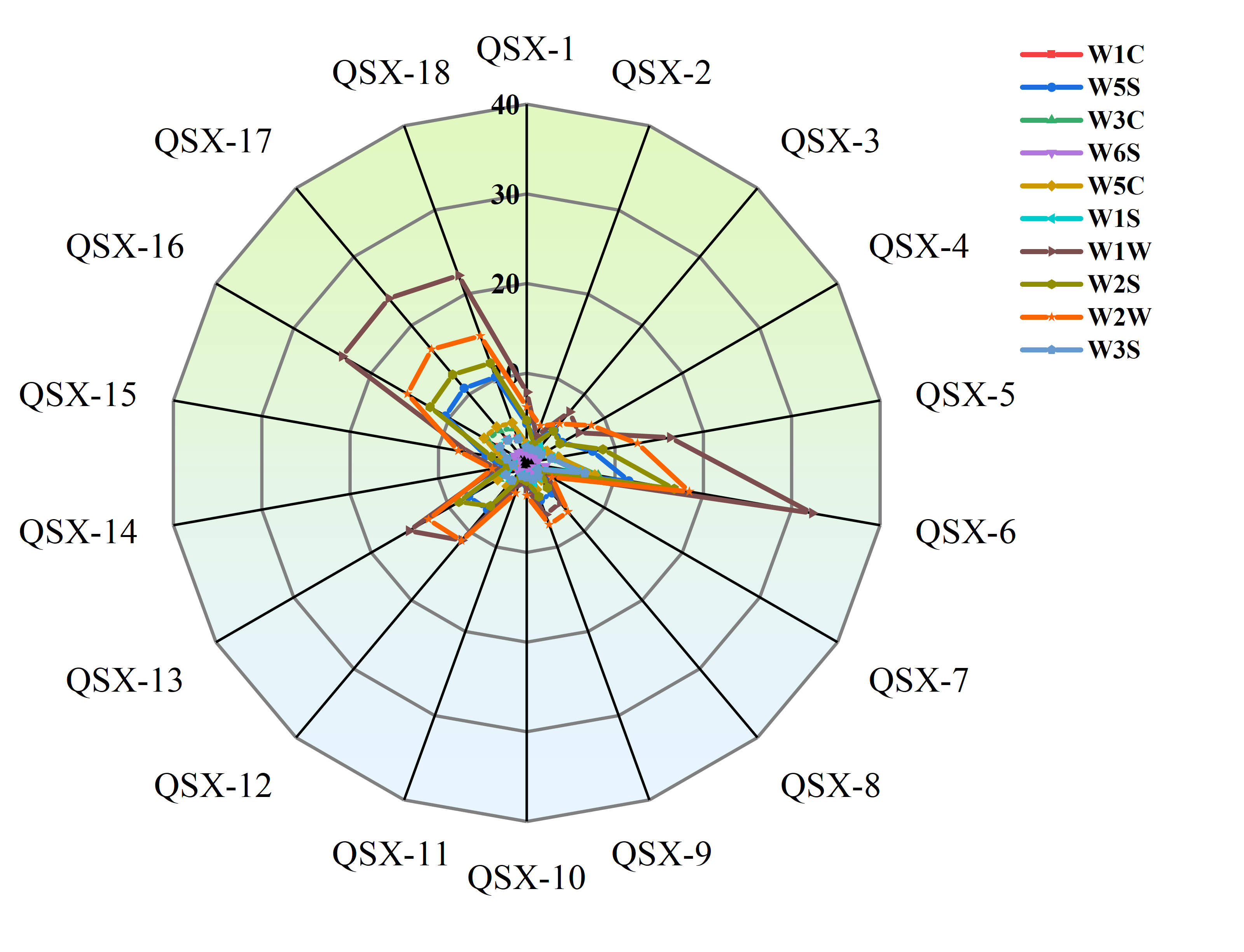 | 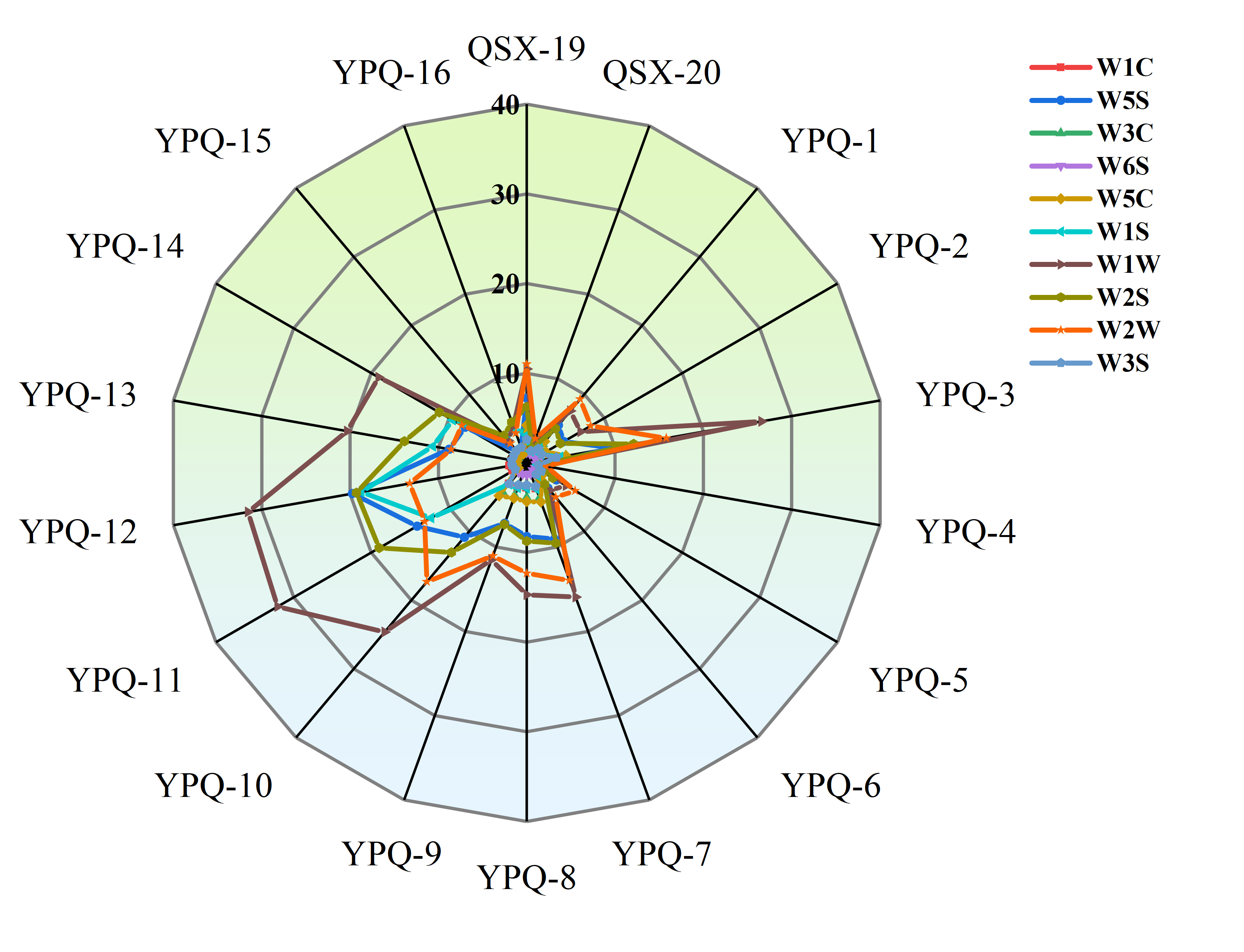 |
| --- | --- |
| 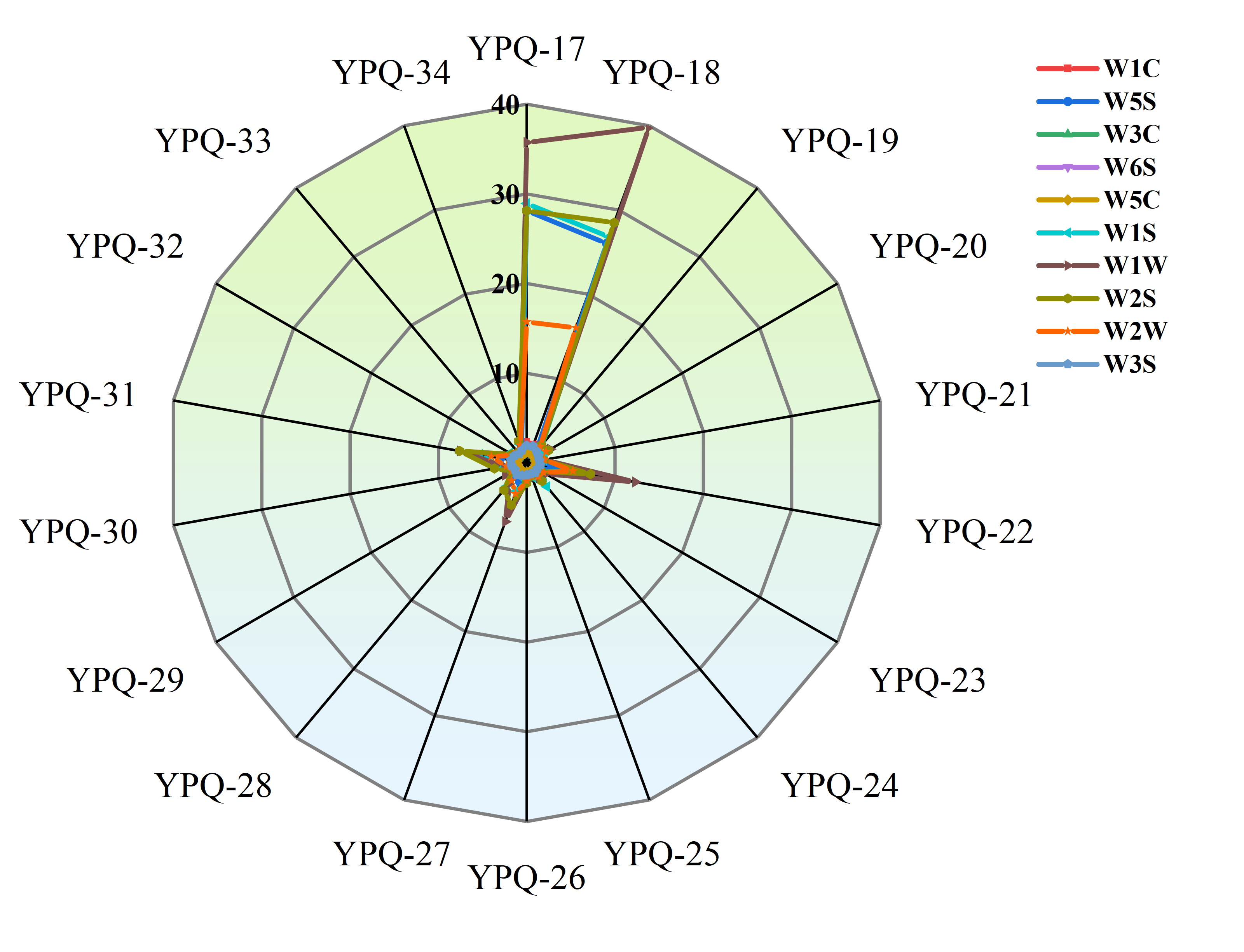 | 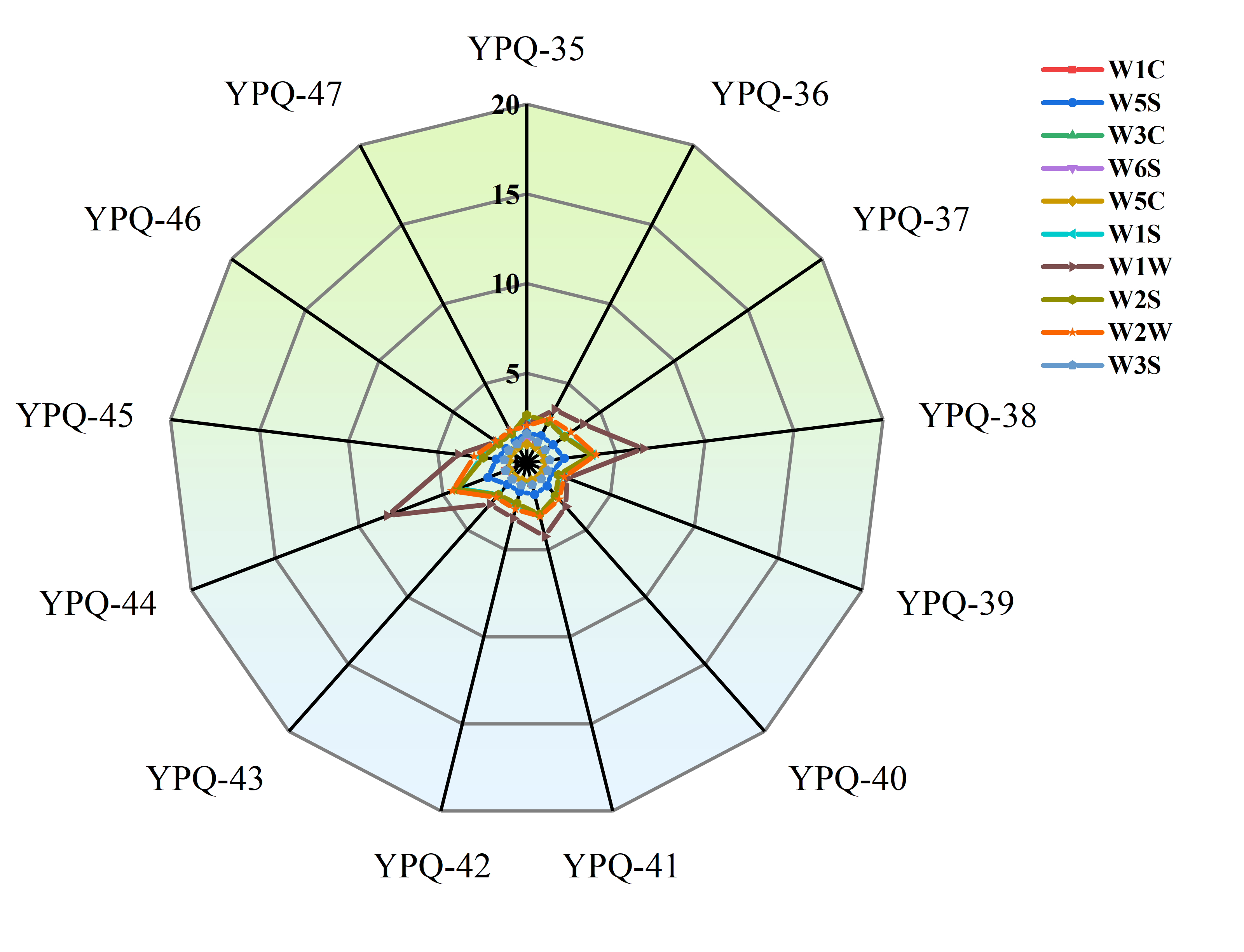 |

| 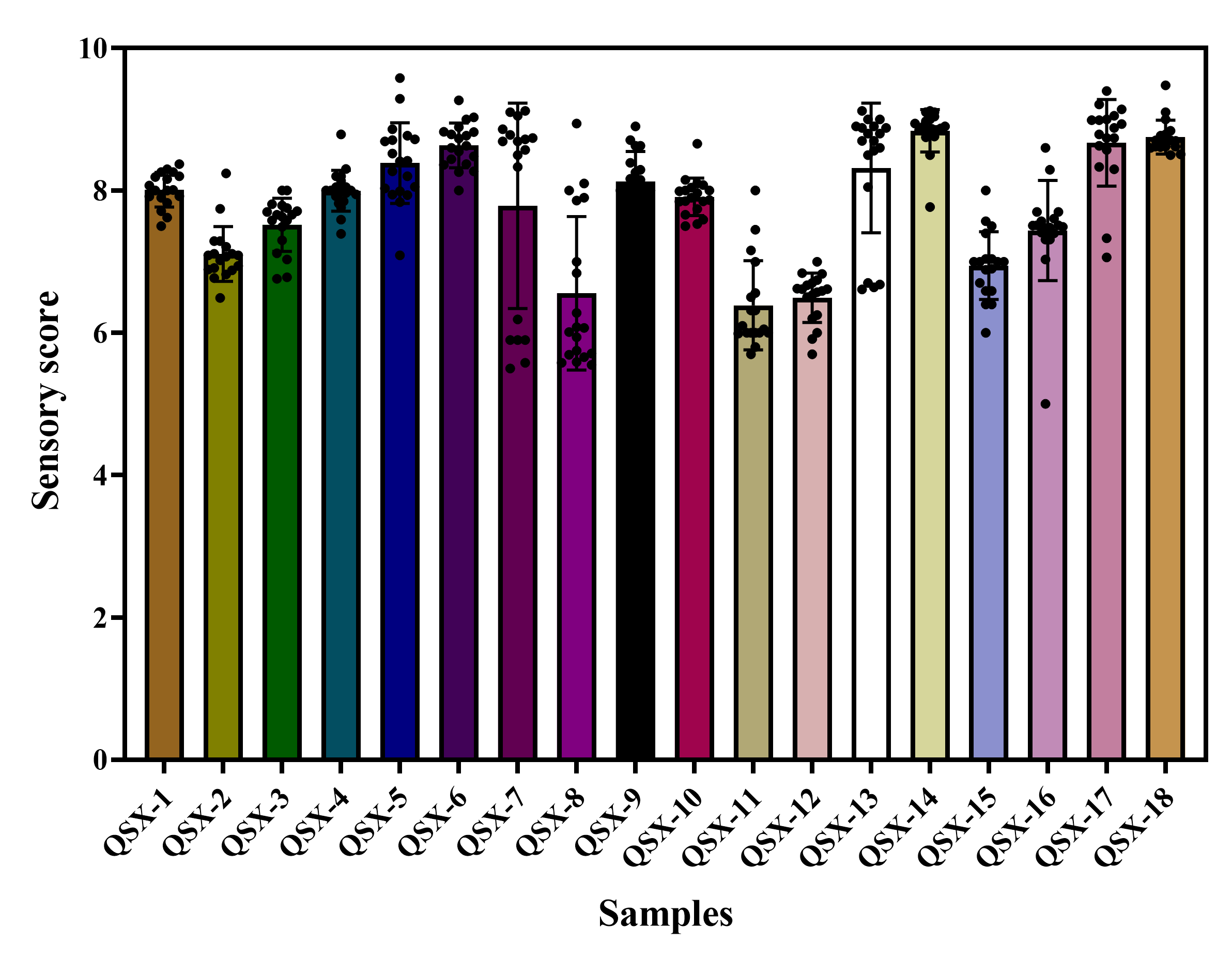 | 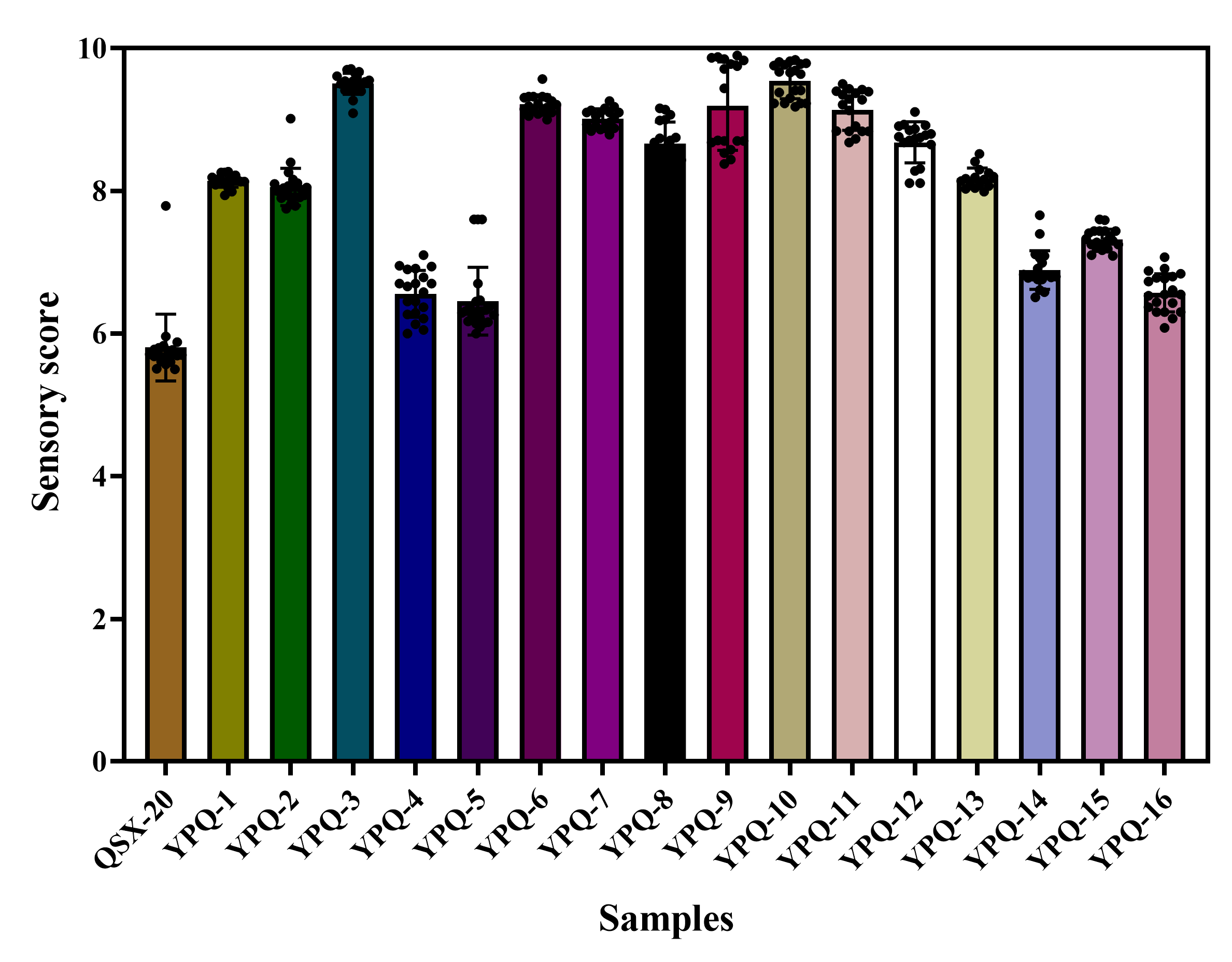 |
| --- | --- |
| 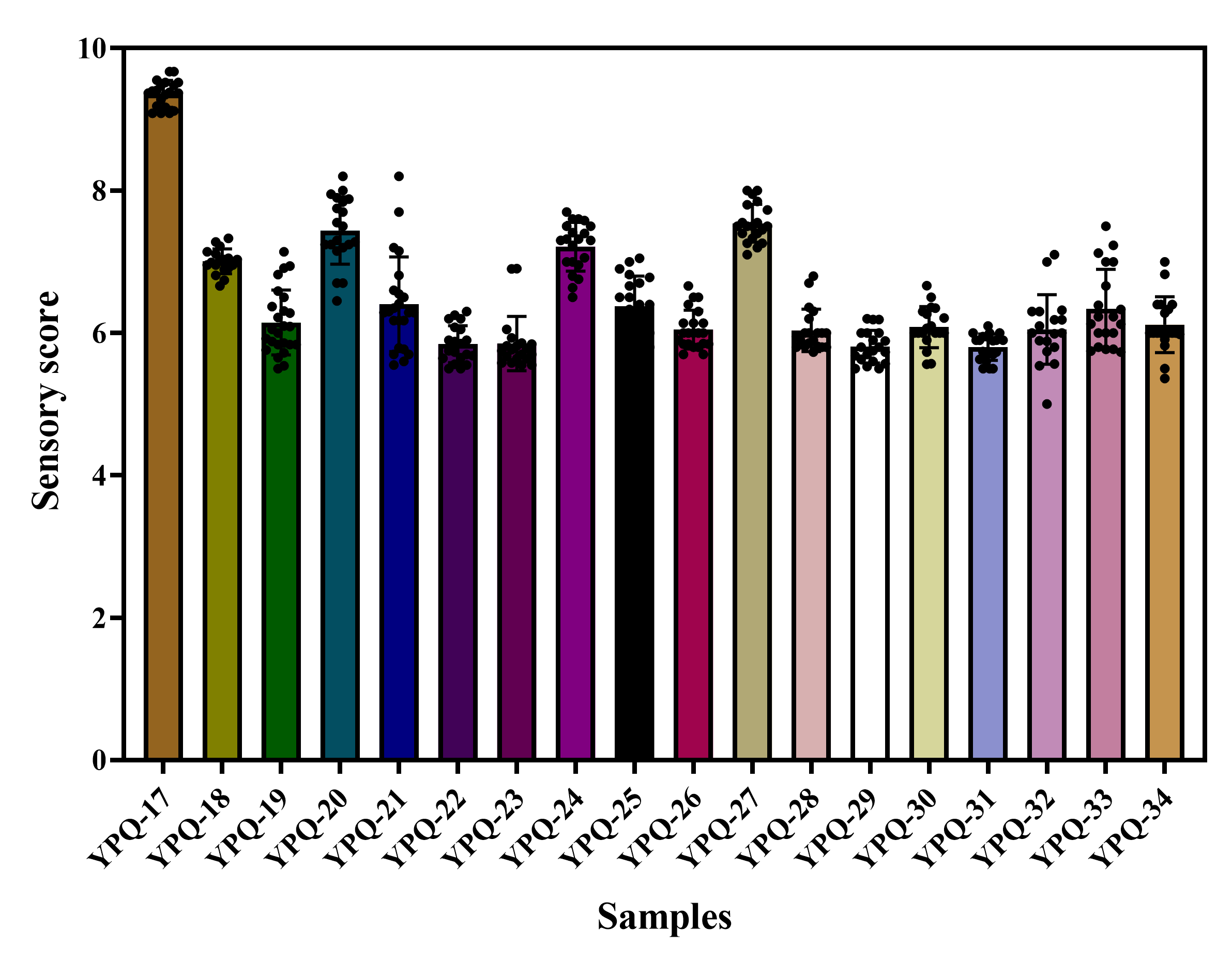 | 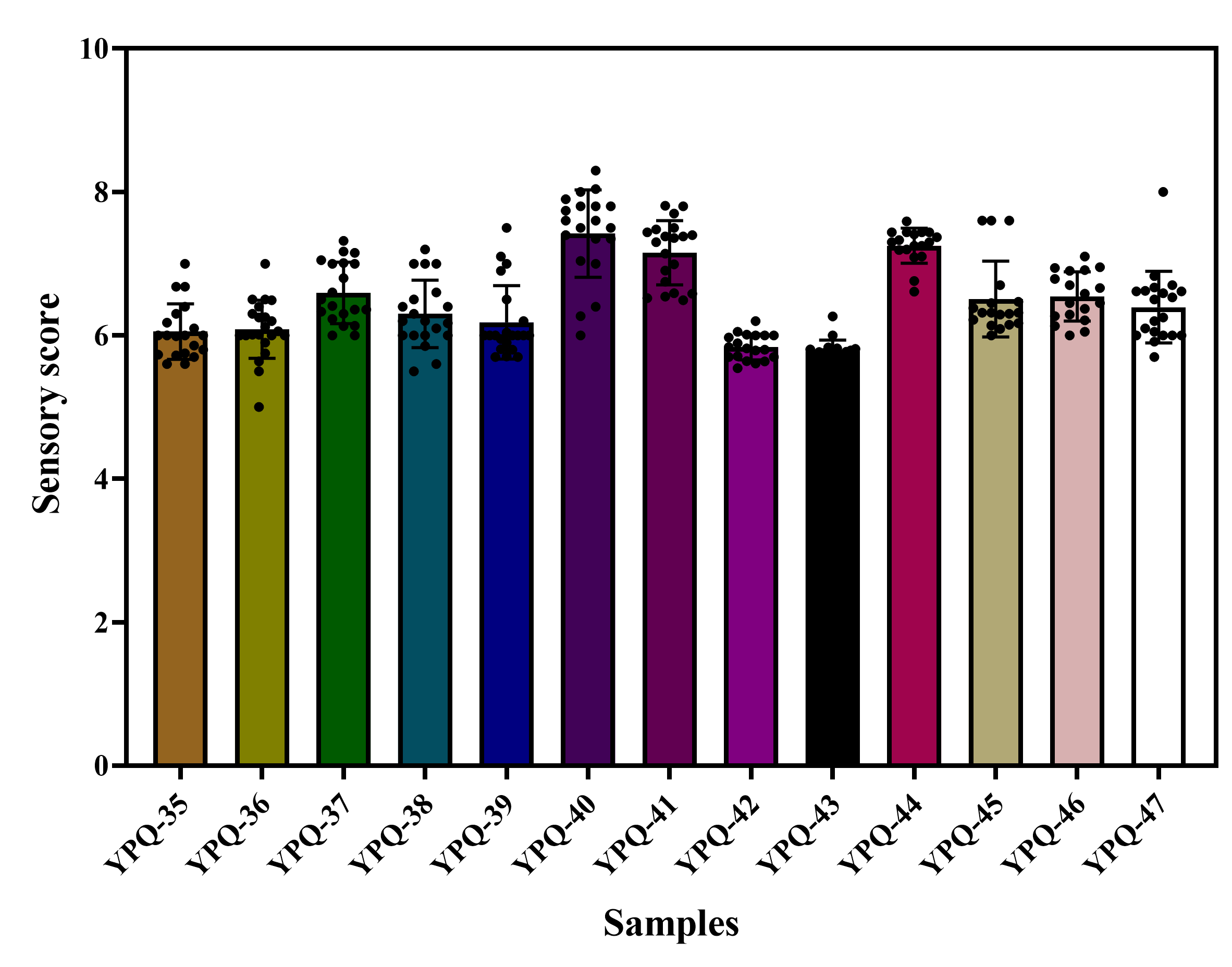 |

**Figure. S1. E-nose and panel sensory score dataset for sensory prediction model construction.
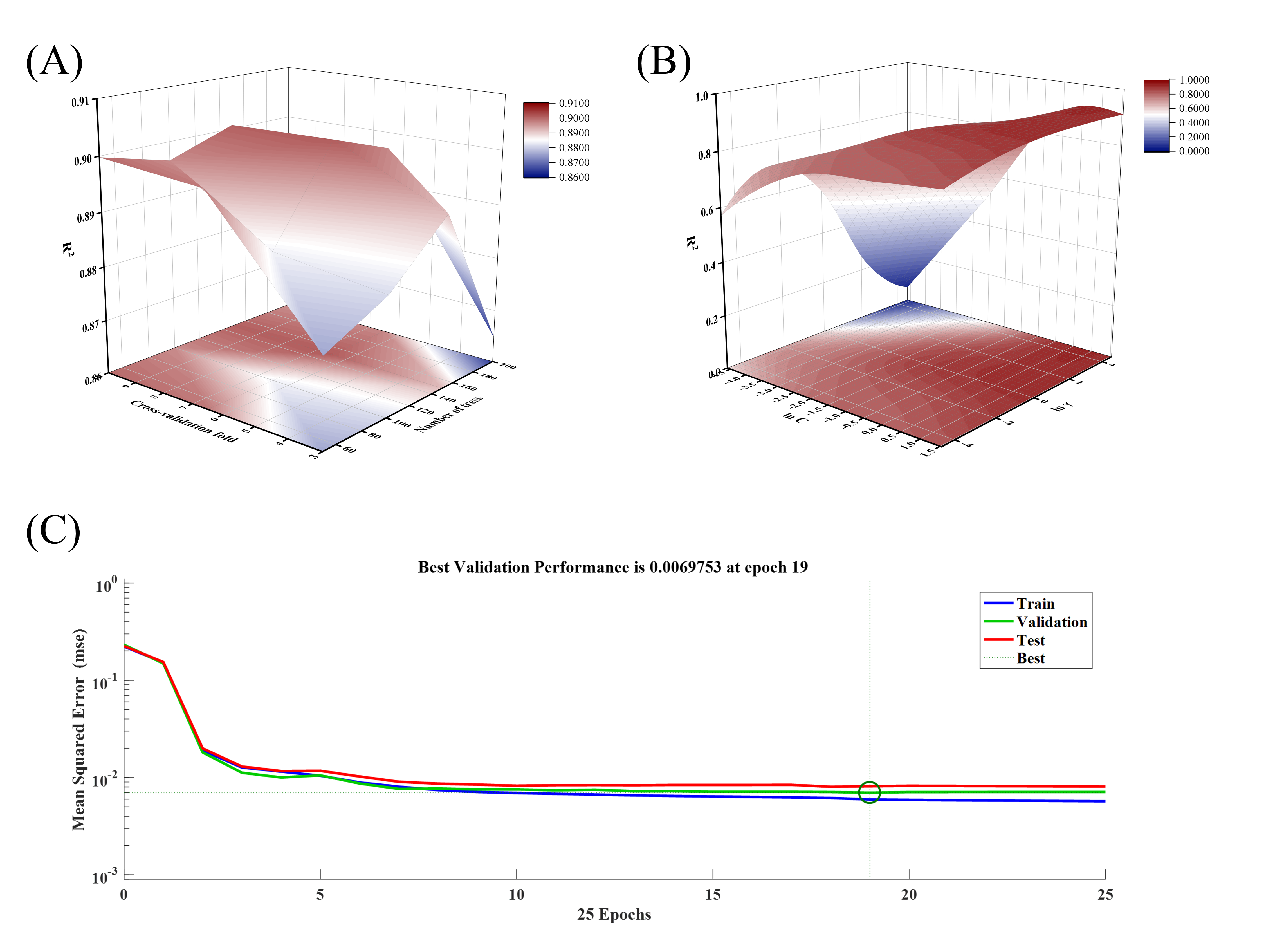
**

**Figure. S2. 3D surface plots of parameter optimization for Random Forest (A) and Support Vector Machine (B), and training performance plot for the back propagation neural network model (C).**


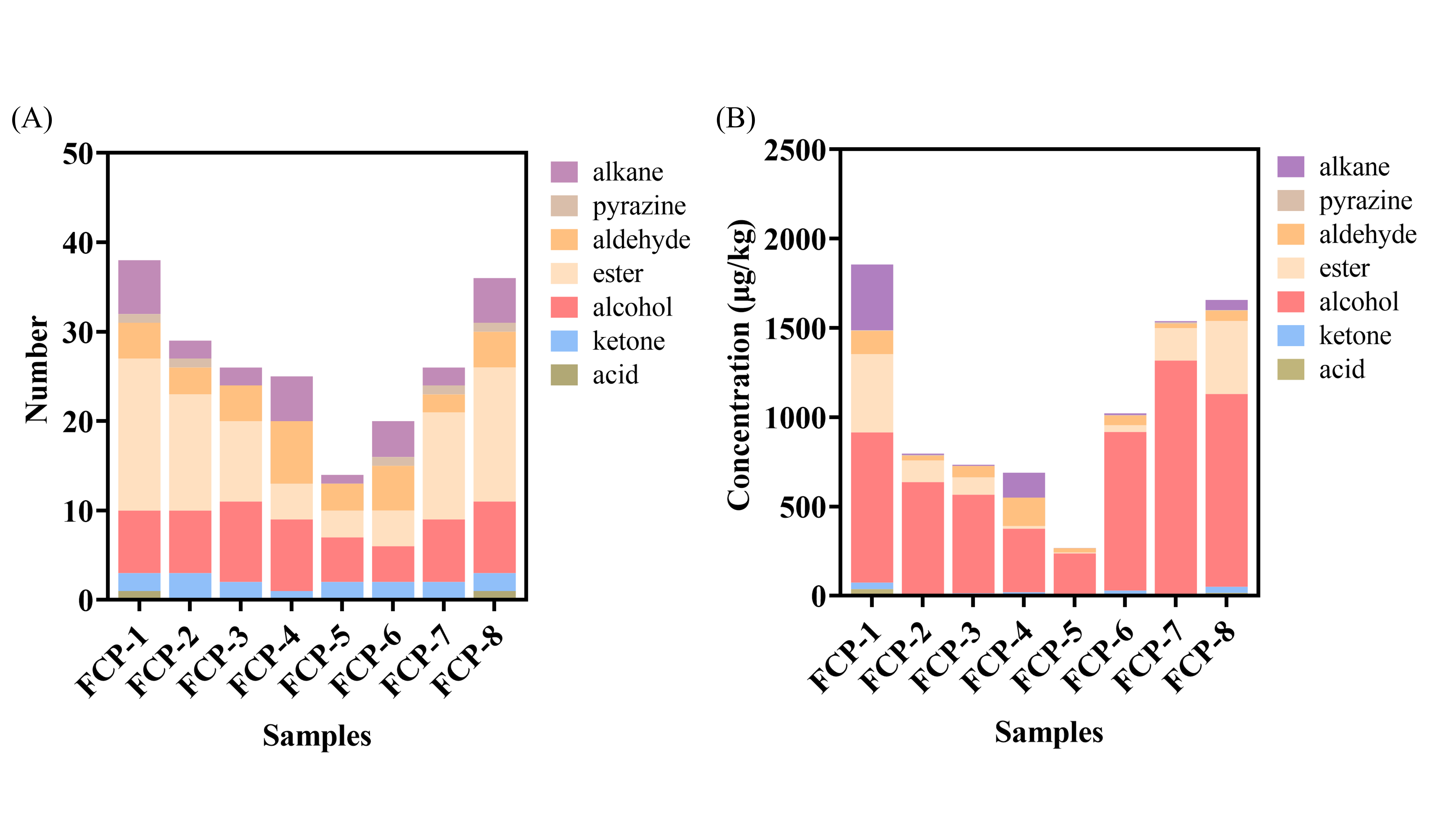


Figure.S3. Stacked bar graph of volatile compounds Number (A), and Concentration (B) in the eight types of ferment chopped pepper.

**Table S1** **The eight samples of fermented chopped peppers from different regions.**

| Samples | Regions |
| --- | --- |
| FCP-1 | Changsha, Hunan, China |
| FCP-2 | Yueyang, Hunan, China |
| FCP-3 | Kunming, Yunan, China |
| FCP-4 | Zunyi, Guizhou, China |
| FCP-5 | Heze, Shandong, China |
| FCP-6 | Zhuzhou, Hunan, China |
| FCP-7 | Zhengzhou, Henan, China |
| FCP-8 | Xiangtan, Huan, China |

**Table S2 Varieties and regions of fermented chopped pepper samples**

| Samples | Peppery varieties | | Regions | Samples | Peppery varieties | Regions | Samples | Peppery varieties | Regions |
| --- | --- | --- | --- | --- | --- | --- | --- | --- | --- |
| YPQ-1 | Line pepper | Xiangtan, Huan, China | | YPQ-24 | Skyward-facing pepper | Xiangtan, Huan, China | YPQ-47 | Skyward-facing pepper | Yiyang, Hunan, China |
| YPQ-2 | Line pepper | Xiangtan, Huan, China | | YPQ-25 | Skyward-facing pepper | Xiangtan, Huan, China | QSX-1 | Line pepper | Changsha, Hunan, China |
| YPQ-3 | Line pepper | Xiangxi, Hunan, China | | YPQ-26 | Skyward-facing pepper | Xiangxi, Hunan, China | QSX-2 | Line pepper | Changsha, Hunan, China |
| YPQ-4 | Line pepper | Jishou, Hunan, China | | YPQ-27 | Skyward-facing pepper | Jishou, Hunan, China | QSX-3 | Line pepper | Changsha, Hunan, China |
| YPQ-5 | Line pepper | Yueyang, Hunan, China | | YPQ-28 | Skyward-facing pepper | Yueyang, Hunan, China | QSX-4 | Line pepper | Changsha, Hunan, China |
| YPQ-6 | Line pepper | Shaoyang, Hunan, China | | YPQ-29 | Skyward-facing pepper | Shaoyang, Hunan, China | QSX-5 | Line pepper | Changsha, Hunan, China |
| YPQ-7 | Line pepper | Shaoyang, Hunan, China | | YPQ-30 | Skyward-facing pepper | Shaoyang, Hunan, China | QSX-6 | Line pepper | Changsha, Hunan, China |
| YPQ-8 | Line pepper | Loudi, Hunan, China | | YPQ-31 | Skyward-facing pepper | Loudi, Hunan, China | QSX-7 | Line pepper | Changsha, Hunan, China |
| YPQ-9 | Line pepper | Changsha, Hunan, China | | YPQ-32 | Skyward-facing pepper | Changsha, Hunan, China | QSX-8 | Line pepper | Changsha, Hunan, China |
| YPQ-10 | Line pepper | Zhuzhou, Hunan, China | | YPQ-33 | Skyward-facing pepper | Zhuzhou, Hunan, China | QSX-9 | Line pepper | Changsha, Hunan, China |
| YPQ-11 | Line pepper | Zhuzhou, Hunan, China | | YPQ-34 | Skyward-facing pepper | Zhuzhou, Hunan, China | QSX-10 | Line pepper | Changsha, Hunan, China |
| YPQ-12 | Line pepper | Chenzhou, Hunan, China | | YPQ-35 | Skyward-facing pepper | Chenzhou, Hunan, China | QSX-11 | Skyward-facing pepper | Changsha, Hunan, China |
| YPQ-13 | Line pepper | Chenzhou, Hunan, China | | YPQ-36 | Skyward-facing pepper | Chenzhou, Hunan, China | QSX-12 | Skyward-facing pepper | Changsha, Hunan, China |
| YPQ-14 | Line pepper | Hengyang, Hunan, China | | YPQ-37 | Skyward-facing pepper | Hengyang, Hunan, China | QSX-13 | Skyward-facing pepper | Changsha, Hunan, China |
| YPQ-15 | Line pepper | Hengyang, Hunan, China | | YPQ-38 | Skyward-facing pepper | Hengyang, Hunan, China | QSX-14 | Skyward-facing pepper | Changsha, Hunan, China |
| YPQ-16 | Line pepper | Changdei, Hunan, China | | YPQ-39 | Skyward-facing pepper | Changdei, Hunan, China | QSX-15 | Skyward-facing pepper | Changsha, Hunan, China |
| YPQ-17 | Line pepper | Changdei, Hunan, China | | YPQ-40 | Skyward-facing pepper | Changdei, Hunan, China | QSX-16 | Skyward-facing pepper | Changsha, Hunan, China |
| YPQ-18 | Line pepper | Loudi, Hunan, China | | YPQ-41 | Skyward-facing pepper | Loudi, Hunan, China | QSX-17 | Skyward-facing pepper | Changsha, Hunan, China |
| YPQ-19 | Line pepper | Changsha, Hunan, China | | YPQ-42 | Skyward-facing pepper | Changsha, Hunan, China | QSX-18 | Skyward-facing pepper | Changsha, Hunan, China |
| YPQ-20 | Line pepper | Yongzhou, Hunan, China | | YPQ-43 | Skyward-facing pepper | Yongzhou, Hunan, China | QSX-19 | Skyward-facing pepper | Changsha, Hunan, China |
| YPQ-21 | Line pepper | Yueyang, Hunan, China | | YPQ-44 | Skyward-facing pepper | Yueyang, Hunan, China | QSX-20 | Skyward-facing pepper | Changsha, Hunan, China |
| YPQ-22 | Line pepper | Yueyang, Hunan, China | | YPQ-45 | Skyward-facing pepper | Yueyang, Hunan, China | YPQ-46 | Skyward-facing pepper | Yiyang, Hunan, China |
| YPQ-23 | Line pepper | Yiyang, Hunan, China | |  |  |  |  |  |  |

**Table S3 Sensors used in PEN3 e-nose and their performance description**

| Sensors | Responsive compounds |
| --- | --- |
| W1C | Sensitive to aromatic compounds |
| W5S | Sensitive to nitrogen oxides |
| W3C | Sensitive to ammonia and aromatic compounds |
| W6S | Sensitive to hydrogens |
| W5C | Sensitive to olefin and aromatic compounds |
| W1S | Sensitive to hydrocarbons |
| W1W | Sensitive to sulfides and pyrazine |
| W2S | Sensitive to alcohols, aldehydes, and ketones |
| W2W | Sensitive to organic sulfides |
| W3S | Sensitive to long-chain alkanes |

**Table S4 Volatile compounds concentration (mean ± standard deviation) in eight types of fermented chopped pepper using GC-MS.**

| Compounds | Identification^a^ | FCP-1 | FCP-2 | FCP-3 | FCP-4 | FCP-5 | FCP-6 | FCP-7 | FCP-8 |
| --- | --- | --- | --- | --- | --- | --- | --- | --- | --- |
| Acetoin | MS, RI | 36.11 ± 14.09^a^ | 5.64 ± 1.66^a^ | 0 | 0 | 0 | 0 | 2.70 ± 2.70^b^ | 32.54 ± 3.05^b^ |
| 3-Methyl-1-butanol | MS, RI | 113.85 ± 67.27^de^ | 260.78 ± 124.53^bc^ | 150.60 ± 34.59^cd^ | 50.14 ± 10.27^de^ | 19.71 ± 1.71^e^ | 7.49 ± 1.10^e^ | 606.21 ± 68.69^a^ | 348.61 ± 15.32^b^ |
| Ethyl isobutyrate | MS, RI | 0.64 ± 0.15^b^ | 0 | 0 | 0 | 0 | 0 | 2.07±2.07^a^ | 0.62±0.88^b^ |
| 1-Pentanol | MS, RI | 8.13±2.78^b^ | 0 | 0 | 23.39 ± 1.01^a^ | 0 | 0 | 0 | 0 |
| Hexanal | MS, RI | 14.2 1± 4.30^c^ | 4.03 ± 0.65^de^ | 25.20 ± 4.65^b^ | 74.91 ± 7.69^a^ | 12.09 ± 3.62^cd^ | 30.86 ± 5.81^b^ | 1.24 ± 1.24^e^ | 15.24 ± 0.43^c^ |
| 1-Hexanol | MS, RI | 177.59 ± 41.44^a^ | 108.11 ± 16.35^bc^ | 67.20 ± 18.61^cd^ | 40.12 ± 4.83^d^ | 25.65 ± 2.97^d^ | 46.92 ± 13.38^d^ | 121.88 ± 17.78^b^ | 184.25 ± 30.55^a^ |
| Isoamyl acetate | MS, RI | 56.05 ± 9.83^ab^ | 45.94 ± 5.41^b^ | 13.65 ± 6.20^c^ | 0 | 0 | 0 | 57.60 ± 6.58^ab^ | 63.35 ± 9.94^a^ |
| Heptaldehyde | MS, RI | 10.05 ± 4.18^a^ | 0 | 0 | 12.89 ± 1.09^a^ | 0 | 3.21 ± 0.18^bc^ | 0 | 4.92 ± 3.71^c^ |
| Ethyl Hexanoate | MS, RI | 32.97 ± 19.79^a^ | 16.75 ± 3.58^ab^ | 14.28 ± 2.71^ab^ | 0 | 0 | 0 | 0 | 30.70 ± 21.85^a^ |
| Hexyl acetate | MS, RI | 5.79 ± 0.96^a^ | 0.19 ± 0.19^b^ | 0 | 0 | 0 | 0 | 0.50 ± 0.50^b^ | 4.94 ± 1.73^a^ |
| Octanal | MS, RI | 22.97 ± 9.02^a^ | 2.49 ± 2.49^bc^ | 7.53 ± 1.41^bc^ | 8.79 ± 1.52^b^ | 0 | 0.48 ± 0.35^c^ | 0 | 8.16 ± 1.49^bc^ |
| Dipentene | MS, RI | 3.16 ± 0.45^bcd^ | 4.06 ± 0.31^bc^ | 3.36 ± 0.83^bcd^ | 1.89 ± 0.19^d^ | 2.09 ± 0.19^d^ | 9.79 ± 1.90^a^ | 4.65 ± 0.23^b^ | 2.48 ± 0.73^cd^ |
| Linalool | MS, RI | 147.68 ± 42.44^cd^ | 221.87 ± 22.57^c^ | 155.81 ± 49.35^cd^ | 97.49 ± 5.20^d^ | 127.02 ± 22.33^d^ | 651.25 ± 36.68^a^ | 380.20 ± 49.19^b^ | 159.20 ± 37.78^cd^ |
| 1-Nonanal | MS, RI | 84.58 ± 35.51^a^ | 20.11 ± 1.89^b^ | 22.58 ± 2.54^b^ | 18.40 ± 0.04^b^ | 2.14 ± 2.14^c^ | 0 | 23.28 ± 3.70^b^ | 30.61 ± 6.78^b^ |
| Phenethyl alcohol | MS, RI | 126.15 ± 55.12^a^ | 10.48 ± 1.51^bc^ | 0 | 0 | 0 | 0 | 98.79 ± 31.27^ab^ | 108.50 ± 83.45^a^ |
| 3-Methoxy-2-isobutylpyrazine | MS, RI | 1.61 ± 1.32^cd^ | 4.16 ± 0.30^b^ | 0 | 0 | 0 | 1.11 ± 0.91^cd^ | 7.06 ± 0.65^a^ | 3.08 ± 0.53^c^ |
| α-Terpineol | MS, RI | 33.39 ± 9.16^c^ | 0 | 52.38 ± 11.59^c^ | 37.51 ± 10.01^c^ | 44.46 ± 11.14^c^ | 182.77 ± 25.41^a^ | 100.01 ± 19.44^b^ | 29.05 ± 6.36^cd^ |
| Ethyl caprylate | MS, RI | 2.95 ± 4.17^a^ | 2.15 ± 2.15^b^ | 0 | 0 | 0 | 0 | 0 | 0 |
| Hexyl 2-methylbutyrate | MS, RI | 29.09 ± 5.88^b^ | 5.16 ± 0.64^c^ | 0 | 0 | 0 | 0 | 22.35 ± 2.17^b^ | 47.58 ± 8.08^a^ |
| Hexyl isovalerate | MS, RI | 17.90 ± 7.73^a^ | 2.92 ± 0.90^bc^ | 0 | 0 | 0 | 3.66 ± 2.63^bc^ | 10.04 ± 0.13^b^ | 19.32 ± 2.89^a^ |
| Ethyl nonanoate | MS, RI | 35.30 ± 7.51^a^ | 7.61 ± 0.72^bc^ | 16.79 ± 5.21^b^ | 0 | 0 | 0 | 12.23 ± 2.12^bc^ | 39.89 ± 11.05^a^ |
| Ethyltrans-4-Decenoate | MS, RI | 131.82 ± 36.02^a^ | 9.83 ± 0.03^b^ | 6.10 ± 6.10^b^ | 6.07 ± 6.07^b^ | 0 | 0 | 23.60 ± 2.46^b^ | 61.30 ± 61.12^b^ |
| Ethyl caprate | MS, RI | 47.01 ± 13.75^a^ | 0 | 5.79 ± 5.79^b^ | 0 | 0 | 0 | 0 | 52.70 ± 14.20^a^ |
| α-Longipinene | MS, RI | 20.91 ± 7.89^a^ | 0 | 0 | 0 | 0 | 0 | 0 | 17.78 ± 3.93^a^ |
| β-Elemene | MS, RI | 2.71 ± 2.05^ab^ | 0 | 0 | 6.81 ± 6.81^a^ | 0 | 0.53 ± 0.75^b^ | 0 | 0.99 ± 1.40^b^ |
| Himachalane | MS, RI | 33.71 ± 5.69^a^ | 0 | 0 | 21.11 ± 21.11^a^ | 0 | 0.40 ± 0.56^b^ | 0 | 33.47 ± 3.86^a^ |
| Ethyl pentadecanoate | MS, RI | 6.12 ± 2.32^b^ | 0 | 4.08 ± 1.81^bc^ | 0 | 0 | 0 | 10.36 ± 4.33^a^ | 4.75 ± 1.30^b^ |
| Eremophylene | MS, RI | 70.63 ± 86.3^a^ | 0 | 0 | 13.32 ± 13.32^b^ | 0 | 0 | 0 | 0 |
| γ-Himachalene | MS, RI | 237.89 ± 192.94^a^ | 0 | 3.6 5± 0.16^b^ | 0 | 0 | 0 | 0 | 0 |
| Ethyl 2-methylbutyrate | MS, RI | 33.78 ± 15.74^a^ | 9.25 ± 2.22^c^ | 11.56 ± 2.79^bc^ | 1.54 ±0^c^ | 0.80 ±0.26^c^ | 0 | 8.92 ± 1.12^c^ | 24.55 ± 5.35^ab^ |
| Palmitic acid ethyl ester | MS, RI | 9.36 ± 4.18^bcd^ | 1.62 ± 1.62^cd^ | 16.37 ± 6.73^b^ | 6.56 ± 6.56^bcd^ | 0 | 4.65 ± 0.78^cd^ | 36.21 ± 9.81^a^ | 11.25 ± 2.88^b^ |
| 4-Methyl-1-pentanol | MS, RI | 234.37 ± 79.14^a^ | 21.82 ± 21.82^bc^ | 51.70 ± 13.91^bc^ | 76.87 ± 6.17^b^ | 0 | 0 | 1.93 ± 1.93^c^ | 234.67 ± 24.82^a^ |
| Ethyl isovalerate | MS, RI | 20.59 ± 5.15^a^ | 4.13 ± 0.99^bc^ | 8.18 ± 2.72^b^ | 0 | 1.54 ± 0.51^c^ | 0.63 ± 0.15^c^ | 5.52 ± 0.62^bc^ | 22.63 ± 4.38^a^ |
| 2-Methyl-2-pentenoic acid | MS, RI | 36.77 ± 26.44^a^ | 0 | 0 | 0 | 0 | 0 | 0 | 15.41 ± 21.80^b^ |
| Ethyl lactate | MS, RI | 6.25 ± 8.84^b^ | 14.78 ± 2.38^a^ | 0 | 0 | 0 | 0 | 0 | 20.53 ± 0.34^a^ |
| 3-Heptanone | MS, RI | 0.46 ± 0.65^de^ | 0.91 ± 0.16^d^ | 1.32 ± 0.30^bc^ | 0 | 2.05 ± 0.34^b^ | 3.97 ± 0.36^a^ | 0 | 1.45 ± 0.33^bc^ |
| Ethyl 2-hydroxybenzoate | MS, RI | 1.88 ± 2.66^b^ | 0 | 0 | 0 | 0 | 28.54 ± 0.96^a^ | 0 | 3.64 ± 2.60^b^ |
| Butyl acetate | MS, RI | 0.97 ± 0.71^b^ | 0 | 0 | 0 | 0 | 0 | 3.36 ± 0.63^a^ | 0 |
| 3-Hexanol | MS, RI | 0 | 2.30 ± 2.30^c^ | 2.02 ± 2.02^c^ | 0 | 8.79 ± 1.17^a^ | 0 | 1.33 ± 1.33^c^ | 5.80 ± 0.04^b^ |
| (E)-3-Hexenol | MS, RI | 0 | 0 | 3.69 ± 3.69^b^ | 0 | 0 | 0 | 0 | 10.67 ± 15.09^a^ |
| (E)-Ocimene | MS, RI | 0 | 4.05 ± 0.29^a^ | 0 | 0 | 0 | 0.21 ± 0.29^c^ | 2.07 ± 2.07^b^ | 2.09 ± 2.95^b^ |
| 4-Methyl-3-pentenol | MS, RI | 0 | 0 | 10.70 ± 0^b^ | 25.33 ± 3.34^a^ | 0 | 0 | 0 | 0 |
| Isobutyl acetate | MS, RI | 0 | 1.31 ± 1.31^a^ | 0 | 0 | 0 | 0 | 0 | 0.62 ± 0.88^b^ |
| 2-Heptanol,3-methyl | MS, RI | 0 | 3.18 ± 3.18^b^ | 27.95 ± 7.35^a^ | 0 | 0 | 0 | 0 | 0 |
| β-Damascenone | MS, RI | 0 | 0.77 ± 0.77^b^ | 0 | 0 | 0 | 0 | 4.35 ± 0.66^a^ | 0 |
| Ethyl vinyl ketone | MS, RI | 0 | 0 | 12.47 ± 2.38^b^ | 19.16 ± 1.04^ab^ | 8.98 ± 2.31^bc^ | 24.93 ± 13.20^a^ | 0 | 0 |
| Valeraldehyde | MS, RI | 0 | 0 | 9.12 ± 5.14^bc^ | 23.32 ± 4.75^a^ | 13.48 ± 4.45^b^ | 17.53 ± 8.11^ab^ | 0 | 0 |
| β-Chamigrene | MS, RI | 0 | 0 | 0 | 96.88 ± 48.37^a^ | 0 | 0 | 0 | 0 |
| Leaf alcohol | MS, RI | 0 | 0 | 0 | 5.13 ± 5.13^a^ | 0 | 0 | 0 | 0 |
| (E)-2-Octenal | MS, RI | 0 | 0 | 0 | 9.64 ± 2.27^a^ | 0 | 3.14 ± 0.87^a^ | 0 | 0 |
| (E)-2-pentenal | MS, RI | 0 | 0 | 0 | 10.99 ± 1.93^a^ | 0 | 0 | 0 | 0 |
| Methyl butyrate | MS, RI | 0 | 0 | 0 | 1.23 ± 1.23^b^ | 3.90 ± 0.69^a^ | 0 | 0 | 0 |

^a^ MS, compounds were identified by MS spectra; RI, compounds were identified by a comparison to the pure standard.

**Table S5 Volatile compounds concentration (mean ± standard deviation) in FCP-1 using GC×GC-O-Q-TOF-MS.**

| RT | | RI^a^ | Compounds | Aroma description | Concentration(μg/kg) | Identification |
| --- | --- | --- | --- | --- | --- | --- |
| 1D | 2D |  |  |  |  |  |
| 6.6093 | 0.698 | 736 | 3-Methyl-1-butanol |  | 515.65 ± 15.65 | MS, RI |
| 8.7095 | 2.149 | 815 | Ethyl lactate |  | 1.20 ± 0.10 | MS, RI |
| 9.4101 | 1.436 | 843 | 4-Methyl-1-pentanol | sour, stink | 125.82 ± 8.96 | MS, RI, aroma |
| 9.9933 | 0.872 | 849 | Ethyl 2-methylbutyrate | banana, pineapple | 79.89 ± 1.77 | MS, RI, aroma |
| 10.5765 | 1.028 | 888 | o-Xylene |  | 10.66 ± 2.12 | MS, RI |
| 10.5765 | 1.755 | 868 | Hexanol |  | 27.70 ± 0.51 | MS, RI |
| 10.8101 | 1.063 | 876 | Isoamyl acetate |  | 31.84 ± 1.27 | MS, RI |
| 11.2767 | 1.358 | 893 | Styrene |  | 37.48 ± 1.44 | MS, RI |
| 11.86 | 3.196 | 907 | Methional | soy sauce | 1.80 ± 0.80 | MS, RI, aroma |
| 13.9601 | 3.676 | 962 | Benzaldehyde |  | 10.32 ± 0.01 | MS, RI |
| 14.1932 | 1.523 | 969 | Ethyl isohexanoate |  | 19.54 ± 0.95 | MS, RI |
| 15.8267 | 1.964 | 1003 | Octanal | fatty | 14.75 ± 1.18 | MS, RI, aroma |
| 16.1764 | 2.074 | 1005 | Ethyl 3-hexenoate |  | 6.12 ± 0.83 | MS, RI |
| 16.2934 | 1.835 | 1011 | Hexyl acetate | refreshing | 8.69 ± 0.03 | MS, RI, aroma |
| 16.9931 | 1.248 | 1029 | β-Limonene |  | 12.69 ± 0.35 | MS, RI |
| 17.1097 | 4.121 | 1036 | Benzyl alcohol |  | 206.91 ± 82.14 | MS, RI |
| 17.5768 | 2.154 | 1041 | Ethyl hex-2-enoate | beer | 16.07 ± 0.03 | MS, RI, aroma |
| 17.5768 | 3.994 | 1045 | Phenylacetaldehyde | refreshing | 59.02 ± 0.79 | MS, RI, aroma |
| 17.8099 | 1.284 | 1037 | β-Ocimene |  | 47.38 ± 2.08 | MS, RI |
| 18.8598 | 3.311 | 1069 | Diethyl malonate |  | 25.05 ± 2.49 | MS, RI |
| 19.6765 | 2.145 | 1074 | Trans-Linalool oxide |  | 72.39 ± 7.63 | MS, RI |
| 19.6765 | 1.537 | 1088 | Terpinolene |  | 8.37 ± 0.50 | MS, RI |
| 19.6765 | 4.011 | 1090 | Guaiacol | pungent | 5.95 ± 0.91 | MS, RI, aroma |
| 20.1432 | 1.703 | 1099 | Linalool | floral | 1163.54 ± 37.60 | MS, RI, aroma |
| 20.3767 | 1.444 | 1101 | Alloxazin |  | 25.74 ± 5.40 | MS, RI |
| 20.3767 | 2.154 | 1107 | Hotrienol |  | 99.65 ± 1.73 | MS, RI |
| 20.4933 | 1.573 | 1108 | Isopentyl Valerate |  | 12.49 ± 0.44 | MS, RI |
| 20.7264 | 1.755 | 1111 | Rose oxide |  | 5.01 ± 1.36 | MS, RI |
| 20.8434 | 3.734 | 1116 | Phenylethyl Alcohol | rose | 1450.79 ± 45.74 | MS, RI, aroma |
| 21.5431 | 1.517 | 1131 | Allocimene B |  | 2.38 ± 0.84 | MS, RI |
| 22.3599 | 3.08 | 1145 | Camphor |  | 4.64 ± 0.48 | MS, RI |
| 22.9431 | 2.789 | 1162 | (E)-2-Nonenal |  | 16.79 ± 7.01 | MS, RI |
| 23.0601 | 3.856 | 1180 | 4-Ethylbenzaldehyde |  | 1.99 ± 0.67 | MS, RI |
| 23.1766 | 4.334 | 1169 | 4-Ethylphenol | stink | 269.61 ± 104.35 | MS, RI, aroma |
| 23.2932 | 2.114 | 1140 | 2-Ethylphenol |  | 8.57 ± 1.43 | MS, RI |
| 23.4098 | 3.278 | 1172 | Ethyl benzoate |  | 98.46 ± 3.56 | MS, RI |
| 23.5267 | 2.452 | 1175 | Levomenthol |  | 10.65 ± 0.59 | MS, RI |
| 23.8764 | 2.44 | 1181 | 3-isobutyl-2-methoxypyrazine | pepper, green | 37.20 ± 2.32 | MS, RI, aroma |
| 23.8764 | 3.37 | 1181 | Diethyl succinate |  | 27.55 ± 3.75 | MS, RI |
| 24.3431 | 2.483 | 1189 | α-Terpineol | clove, woody | 525.12 ± 21.32 | MS, RI, aroma |
| 24.5766 | 3.348 | 1192 | Methyl salicylate |  | 176.52 ± 7.05 | MS, RI |
| 24.5766 | 1.721 | 1196 | Ethyl caprylate |  | 97.51 ± 2.78 | MS, RI |
| 25.0433 | 1.534 | 1206 | 4-Methylpentyl isovalerate |  | 65.77 ± 0.85 | MS, RI |
| 25.0433 | 2.035 | 1206 | Decanal |  | 40.95 ± 1.35 | MS, RI |
| 25.5099 | 2.908 | 1225 | α,4-Dimethyl-3-cyclohexene-1-acetaldehyde |  | 59.43 ± 3.69 | MS, RI |
| 25.6265 | 1.556 | 1224 | 2,3-Dihydrobenzofuran |  | 0.83 ± 0.04 | MS, RI |
| 26.4432 | 1.6 | 1236 | Hexyl 2-methylbutyrate |  | 9.55 ± 0.27 | MS, RI |
| 27.1434 | 3.51 | 1255 | Geraniol |  | 4.73 ± 4.73 | MS, RI |
| 27.1434 | 3.882 | 1228 | Nerol |  | 2.07 ± 0.06 | MS, RI |
| 27.3765 | 3.65 | 1258 | Phenethyl acetate |  | 47.15 ± 3.09 | MS, RI |
| 27.9597 | 3.162 | 1270 | Ethyl 2-hydroxybenzoate |  | 186.69 ± 2.67 | MS, RI |
| 29.01 | 1.762 | 1295 | Ethyl nonanoate |  | 36.11 ± 1.24 | MS, RI |
| 29.2431 | 2.021 | 1302 | Spiroxide | tea-leaf, green | 17.22 ± 2.82 | MS, RI, aroma |
| 29.8268 | 1.599 | 1315 | 4-Methylpentyl 4-methylpentanoate |  | 149.00 ± 3.88 | MS, RI |
| 29.8268 | 4.276 | 1316 | 4-vinylguaiacol |  | 40.25 ± 5.77 | MS, RI |
| 31.1093 | 1.805 | 1347 | Ethyl trans-4-decenoate | fruity | 1549.30 ± 40.67 | MS, RI, aroma |
| 31.4599 | 1.631 | 1353 | α-longipinene |  | 66.66 ± 66.66 | MS, RI |
| 31.3433 | 3.515 | 1353 | Ethyl 3-phenylpropionate | seasoning | 20.47 ± 1.13 | MS, RI, aroma |
| 31.9265 | 4.815 | 1365 | γ-Nonanolactone |  | 7.82 ± 2.69 | MS, RI |
| 32.3932 | 1.574 | 1372 | Ylangene |  | 274.30 ± 4.85 | MS, RI |
| 32.3932 | 2.484 | 1374 | 2,2,4-Trimethyl-3-hydroxypentyl isobutyrate |  | 24.17 ± 0.47 | MS, RI |
| 32.8598 | 3.034 | 1386 | β-Damascenone | sweaty, honey | 26.92 ± 0.58 | MS, RI, aroma |
| 33.2099 | 1.714 | 1391 | β-Elemen |  | 189.04 ± 6.35 | MS, RI |
| 33.6762 | 1.911 | 1419 | Caryophyllene |  | 74.85 ± 74.85 | MS, RI |
| 33.6766 | 3.993 | 1403 | Methyleugenol |  | 2.81 ± 1.04 | MS, RI |
| 33.9101 | 1.947 | 1406 | Longifolene |  | 51.47 ± 8.84 | MS, RI |
| 34.2598 | 2.704 | 1433 | Dihydro-b-ionone |  | 25.99 ± 1.77 | MS, RI |
| 35.8933 | 1.646 | 1494 | Ethyl undecylate |  | 201.31 ± 11.61 | MS, RI |
| 36.1264 | 4.152 | 1463 | Cinnamic acid ethyl ester |  | 1.84 ± 0.34 | MS, RI |
| 36.9432 | 3.138 | 1486 | β-lonone | floral | 232.60 ± 221.00 | MS, RI, aroma |
| 37.7599 | 2.113 | 1500 | Himachalene |  | 378.45 ± 66.45 | MS, RI |
| 38.4601 | 1.936 | 1524 | β-sesquiphellandrene |  | 66.47 ± 17.65 | MS, RI |
| 38.9268 | 2.497 | 1577 | Ent-Spathulenol |  | 23.86 ± 0.93 | MS, RI |
| 39.9766 | 2.091 | 1564 | Nerolidol | fruity | 442.62 ± 3.55 | MS, RI, aroma |
| 41.143 | 1.731 | 1594 | Ethyl laurate |  | 404.22 ± 4.35 | MS, RI |
| 41.7267 | 2.819 | 1585 | (-)-Epiglobulol |  | 2.69 ± 0.20 | MS, RI |
| 41.8432 | 2.076 | 1570 | Cyclododecanol |  | 3.21 ± 3.21 | MS, RI |
| 43.2432 | 1.625 | 1645 | Isoamyl caprate |  | 1.74 ± 1.74 | MS, RI |
| 43.4768 | 1.67 | 1687 | Ethyl tridecanoate |  | 90.29 ± 7.04 | MS, RI |
| 44.1765 | 1.852 | 1680 | 13-Methyltetradecanal |  | 467.23 ± 23.66 | MS, RI |
| 44.5266 | 2.7 | 1684 | α-bisabolol |  | 0.56 ± 0.56 | MS, RI |
| 44.6432 | 1.821 | 1692 | 6-Nonenoic acid, 8-methyl-, 4-methylpentyl ester |  | 204.60 ± 0.16 | MS, RI |
| 45.2264 | 1.681 | 1710 | Nonanoic acid, 8-methyl-, 4-methylpentyl ester |  | 132.98 ±3.85 | MS, RI |
| 46.0431 | 3.749 | 1695 | (1R,7S,E)-7-Isopropyl-4,10-dimethylenecyclodec-5-enol |  | 14.57 ± 0.10 | MS, RI |
| 48.9601 | 1.755 | 1882 | Heptyl caprate |  | 0.64 ± 0.64 | MS, RI |
| 49.3097 | 1.707 | 1825 | Isopropyl myristate |  | 17.53 ± 11.70 | MS, RI |
| 49.6598 | 3.317 | 1846 | Exaltoin |  | 15.43 ± 0.85 | MS, RI |
| 49.7764 | 2.022 | 1865 | 9-pentadecenoic acid, ethyl ester |  | 406.37 ± 0.90 | MS, RI |
| 51.6434 | 1.779 | 1894 | Ethyl Pentadecanoate |  | 169.22 ± 0.47 | MS, RI |
| 52.6933 | 1.912 | 1883 | Methyl isopalmitate |  | 5.08 ± 5.08 | MS, RI |
| 53.0434 | 1.921 | 1976 | Ethyl 9-hexadecenoate |  | 178.04 ± 0.61 | MS, RI |
| 54.7935 | 1.786 | 1993 | Palmitic acid ethyl ester |  | 1958.69 ± 11.26 | MS, RI |
| 55.9599 | 2.073 | 2071 | (Z)-Ethyl heptadec-9-enoate |  | 27.26 ± 0.18 | MS, RI |
| 59.8101 | 2.341 | 2160 | Ethyl linolate |  | 813.1 ± 52.07 | MS, RI |
| 60.0432 | 2.154 | 2174 | Ethyl arachidate |  | 89.22 ± 1.18 | MS, RI |
| 60.0432 | 2.546 | 2169 | Linolenic acid ethyl ester |  | 761.00 ± 31.58 | MS, RI |
| 60.7434 | 1.965 | 2195 | Ethyl stearate |  | 10.76 ± 3.11 | MS, RI |

^a^ MS, compounds were identified by MS spectra; aroma, compounds were identified by the aroma descriptors; RI, compounds were identified by a comparison to the pure standard.
